# No evidence for an effect of selective spatial attention on the development of secondary hyperalgesia: A replication study

**DOI:** 10.3389/fnhum.2022.997230

**Published:** 2022-11-03

**Authors:** Delia Della Porta, Marie-Lynn Vilz, Avgustina Kuzminova, Lieve Filbrich, André Mouraux, Valéry Legrain

**Affiliations:** ^1^Institute of Neuroscience, Université catholique de Louvain, Brussels, Belgium; ^2^Psychological Sciences Research Institute, Université catholique de Louvain, Louvain-la-Neuve, Belgium; ^3^Louvain Bionics, Université catholique de Louvain, Louvain-la-Neuve, Belgium

**Keywords:** central sensitization, selective spatial attention, secondary hyperalgesia, top-down control of attention, replication study, pain, nociception

## Abstract

Central sensitization refers to the increased responsiveness of nociceptive neurons in the central nervous system after repeated or sustained peripheral nociceptor activation. It is hypothesized to play a key role in the development of chronic pain. A hallmark of central sensitization is an increased sensitivity to noxious mechanical stimuli extending beyond the injured location, known as secondary hyperalgesia. For its ability to modulate the transmission and the processing of nociceptive inputs, attention could constitute a promising target to prevent central sensitization and the development of chronic pain. It was recently shown that the experimental induction of central sensitization at both forearms of healthy volunteers using bilateral high-frequency electrocutaneous stimulation (HFS), can be modulated by encouraging participants to selectively focus their attention to one arm, to the detriment of the other arm, resulting in a greater secondary hyperalgesia on the attended arm as compared to the unattended one. Given the potential value of the question being addressed, we conducted a preregistered replication study in a well-powered independent sample to assess the robustness of the effect, i.e., the modulatory role of spatial attention on the induction of central sensitization. This hypothesis was tested using a double-blind, within-subject design. Sixty-seven healthy volunteers performed a task that required focusing attention toward one forearm to discriminate innocuous vibrotactile stimuli while HFS was applied on both forearms simultaneously. Our results showed a significant increase in mechanical sensitivity directly and 20 min after HFS. However, in contrast to the previous study, we did not find a significant difference in the development of secondary hyperalgesia between the attended vs. unattended arms. Our results question whether spatial selective attention affects the development of secondary hyperalgesia. Alternatively, the non-replication could be because the bottom-up capture of attention caused by the HFS-mediated sensation was too strong in comparison to the top-down modulation exerted by the attentional task. In other words, the task was not engaging enough and the HFS pulses, including those on the unattended arm, were too salient to allow a selective focus on one arm and modulate nociceptive processing.

## Introduction

Pain is an unpleasant sensory experience associated with ongoing or potential tissue damage. In normal conditions, pain is evoked by the activation of specific receptors, nociceptors that are characterized by high activation thresholds and thus respond selectively to high intensity and potentially harmful stimuli. The nociceptive system defines the processes involved in coding, transmitting, and processing sensory information conveyed by nociceptors. After a traumatic injury, sensitivity to pain increases in the area where the injury occurred (i.e., primary hyperalgesia), but also in the area surrounding it (i.e., secondary hyperalgesia). This phenomenon, known as sensitization, can be considered as a learning process through which the repeated administration of a noxious stimulus leads to an increased response toward the stimulus itself (Latremoliere and Woolf, 2009). It could serve as protection against further injury and facilitate the healing process by limiting movements and exposure of the injured site (Latremoliere and Woolf, 2009). Whereas primary hyperalgesia mainly results from sensitization of the peripheral nerves, secondary hyperalgesia seems to be a consequence of sensitization at the level of the central nervous system, especially at the level of the first synapse relaying nociceptive input in the dorsal horn of the spinal cord (Woolf, 1983, 2011; Raja et al., 1984; LaMotte et al., 1991; Klede et al., 2003). Evidence from animal studies suggests that central sensitization originates, at least in part, from a mechanism of activity-dependent plasticity, in which dorsal horn circuits undergo a series of functional changes that result behaviorally in an enhanced responsiveness to peripheral stimuli, especially mechano-nociceptive stimuli such as mechanical pinprick stimuli applied onto the skin (Raja et al., 1984; Latremoliere and Woolf, 2009). The activity-dependent plasticity that leads to these functional changes is long-lasting but not permanent. However, for some individuals, this state of central sensitization could perdure far beyond the healing process. It is therefore suggested that, besides its protective and adaptive role, central sensitization could play a key role in the development and the maintenance of chronic pain (Woolf and Salter, 2000; Latremoliere and Woolf, 2009; Woolf, 2011; Harte et al., 2018). Understanding the underlying mechanisms of central sensitization and the elements that trigger these adaptive changes is therefore essential to prevent and treat chronic pain conditions.

Several methods can be used to experimentally induce secondary hyperalgesia in healthy volunteers. One of these is the application of a series of strong peripheral nociceptive stimuli using high-frequency electrical stimulation (HFS) of the skin *via* a multi-pin electrode designed to preferentially activate skin nociceptors. Several studies show that HFS of a few seconds reliably induce an increased sensitivity to mechanical pinprick stimuli applied in the area surrounding the stimulated site (e.g., Klein et al., 2004, 2008; van den Broeke et al., 2010, 2016a,b; Pfau et al., 2011; van den Broeke and Mouraux, 2014). The HFS-induced increase in sensitivity is not observed immediately, but several minutes after HFS (showing that it is not related to HFS-induced ongoing pain sensation) and lasts several hours. While the exact underlying mechanisms are still a matter of debate, HFS-induced secondary hyperalgesia is considered a valid marker of central sensitization (e.g., van den Broeke et al., 2020).

Emotional and cognitive factors such as attention and expectations are increasingly acknowledged to play an important role in the development and maintenance of chronic pain (Crombez et al., 2004, 2005; van Damme et al., 2008, 2010; Legrain et al., 2012; Navratilova and Porreca, 2014; van Ryckeghem et al., 2018). It is hypothesized that changes in sensitivity to pain associated with some chronic pain conditions could involve a top-down modulation operated by cognitive factors. Most importantly, cognitive factors seem to influence the activity of the descending pain modulation system, modifying the spinal transmission of nociceptive inputs, either facilitating or inhibiting pain (Tracey and Mantyh, 2007; Eippert et al., 2009; Sprenger et al., 2012). In support of this hypothesis, studies have suggested that, in healthy volunteers, the development of secondary hyperalgesia can be modulated by the induction of positive or negative expectations (Matre et al., 2006; Salomons et al., 2014; van den Broeke et al., 2014). Torta et al. (2020) demonstrated that secondary hyperalgesia may be reduced when participants are required to perform, concomitantly to the induction of secondary hyperalgesia, a very demanding and difficult cognitive task (Such as a task involving working memory abilities). Similarly, Filbrich et al. (2020) reported that selectively directing spatial attention to one arm can modify the experimental induction of central sensitization in healthy volunteers, leading to an enhanced sensitivity to mechanical pinprick stimuli applied on the arm where attention was focused on, as compared to sensitivity tested on the other arm. To induce secondary hyperalgesia, they simultaneously applied HFS on the left and right forearms while participants performed a task requiring discriminating vibrotactile stimuli specifically delivered on one of the two forearms, while ignoring all the other stimuli on the opposite arm. As both forearms were sensitized at the same time, the effects of the spatial task performed during the induction of secondary hyperalgesia selectively depended on the focus of attention. These findings could represent an important step forward in understating how top-down modulation can selectively affect activity-dependent plasticity of nociceptive pathways, making attention a malleable target for psychological interventions in clinical settings.

However, the results of Filbrich et al. (2020), despite reaching statistical significance, showed very small differences (5 points on a 100-point numerical scale used to assess intensity perception), and the hypothesis was tested in a small sample (*N* = 21), which could lessen the reliability of the results. In low-powered studies, the chance of discovering false-positives increases, and it is more likely that the magnitude of the effect size is inflated (Ioannidis, 2005; Button et al., 2013).

With the present study we aimed to strengthen the evidence surrounding the effect of selective attention on the induction of secondary hyperalgesia, by assessing the robustness of this effect with a well-powered preregistered replication study conducted in a large sample of healthy volunteers.

## Materials and methods

### Participants

Seventy-two participants took part in the experiment. Sample size was determined using G*power for a one-sided paired sample *t*-test considering the smallest effect size found in Filbrich et al. (2020) (*d* = 0.32, α = 0.05 and, power = 0.85). The sample size required was of about 62 participants. To account for attrition, the estimated sample size was increased by 10, resulting in a sample of 72 participants.

Participants were recruited through flyers distributed around the local university campus and through advertisements on social media. They were told that the purpose of the study was to assess performance on a cognitive task while high-frequency stimulation was applied. Exclusion criteria were suffering from severe physical morbidity, neurological or psychiatric disease, traumatic injury of the upper limbs (currently or in the last 6 months), pain complains over the last 6 months, regular use of psychotropic and analgesic drugs, use of a pacemaker, and in case of female participants, pregnancy, or breastfeeding. Having participated in a previous HFS experiment in the lab was also considered as an exclusion criterium. Furthermore, eligible participants were asked to (1) have slept at least 6 h before the experiment, (2) restrain from drinking caffeinated beverages, and (3) not use any alcohol and medication in the 12 h preceding the study. Eligibility was checked in the lab through a digital questionnaire (Qualtrics, Provo, UT, USA; approx. 4 min).

The experimental procedure was approved by the local ethics committee (Commission d’Ethique biomédicale hospital-facultaire, Saint-Luc university Hospital and UCLouvain, N° Enregistrement Belge B403201214265), in agreement with the Declaration of Helsinki. All participants signed informed consent before the experimental session and received financial compensation for their participation.

### Stimuli and apparatus

#### Induction of secondary hyperalgesia at the left and right volar forearms

**FIGURE 1:**
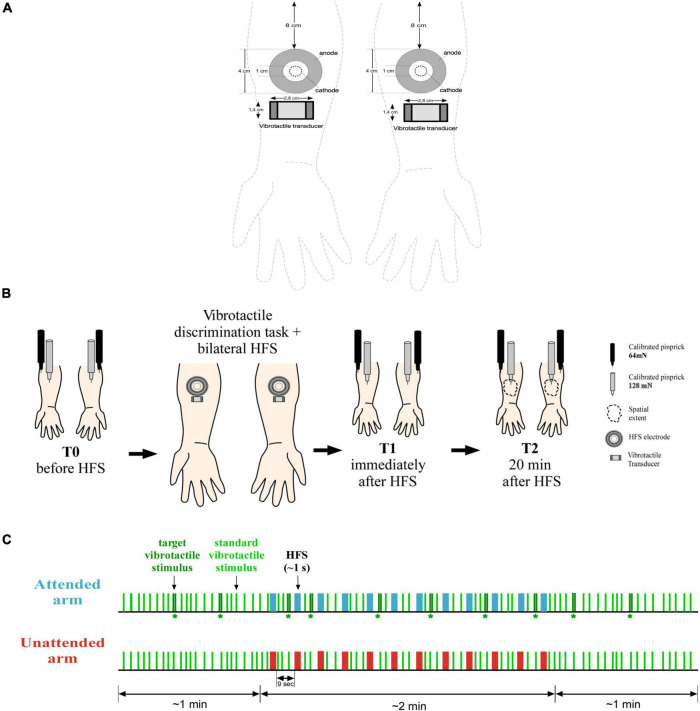
Materials and method. **(A)** HFS was delivered on both arms using two electrodes consisted of 16 blunt stainless-steel pins placed in 10-mm diameter circle (cathode), surrounded by a stainless-steel circular anode. The vibrotactile transducer was placed on the skin immediately below the electrode (ca. 2 cm). Experimental procedure. **(B)** On both forearms, mechanical pinprick sensitivity was measured with two different intensity of pinprick stimulation (128 and 64 mN), before the simultaneous and bilateral delivery of HFS in combination with the detection task (T0), immediately after (T1), and 20 min later (T2). In addition, at time T2, the extent of the area of increased sensitivity was measured along the medial-later and the proximal-distal axes. Manipulation **(C)** 93 standard vibrotactile stimuli (a single 20-ms vibration) were delivered simultaneously on each arm at a random interval between 2 and 5 s. On 10 occasions, a target vibrotactile stimulus (two succeeding vibrations separated by 50-ms) was delivered on the attended arm while the unattended arm received a standard stimulus. During the first minute of the task, only vibrotactile stimuli were delivered, with the occurrence of at least two target stimuli. In the next 2 min, 12 trains of HFS pulses were delivered concomitantly to the task, simultaneously on each arm with an inter-trial interval of 9 s. When a train was delivered, no vibrotactile stimuli were presented, and a target vibrotactile stimulus could be presented only after 2 s from a train. For the last minute, as for the first, only vibrotactile stimuli were delivered, with the presence of at least two target stimuli. Participants were instructed to lift their foot off a pedal every time a target stimulus was delivered. Moreover, they were told to only pay attention to the arm where the target stimuli were delivered, and to ignore all the other stimuli.

High-frequency stimulation (HFS) was used to induce secondary hyperalgesia and was delivered to the skin of both volar forearms using two custom multi-pin surface electrodes designed to preferentially activate skin nociceptors. The electrodes followed the design proposed by the Center for Sensory-Motor Interaction (Aalborg University, Denmark). They consist of 16 blunt stainless-steel pins with a diameter of 0.2 mm protruding 1 mm from the base. The pins are distributed in a circle of 10-mm diameter and serve as cathode. The anode is a stainless-steel circular electrode concentrically located around the pins with 22-mm inner diameter and 40-mm outer diameter. One electrode was placed on each forearm at 8 cm distance from the middle of the cubital fossa (see Figure 1A for more details). Electrical stimulation was generated by two constant current electrical stimulators (DS7A; Digitimer Ltd., Welwyn Garden City, UK) and consisted of 500 square-wave pulses spread in 12 trains (single pulse-width 2 ms) of 100 Hz lasting about 0.42 s each, with an inter-train interval of 9 s (i.e., burst-like stimulation; see Gousset et al., 2020). Total HFS duration was about 2 min. This HFS stimulation pattern (12 trains at 100 Hz) differed slightly from the stimulation pattern used by Filbrich et al. (2020) (5 trains at 100 Hz) and the one described in the preregistration (12 trains at 42 Hz), but the total number of pulses (*N* = 500) and their presentation as stimulation bursts separated by a short interval were identical. We hypothesized that the chosen HFS protocol with an increased number of trains, could potentially facilitate habituation to the HFS stimuli decreasing their salience and consequently, facilitating attentional selectivity toward the attended arm (Legrain et al., 2009a).

The intensity of stimulation was defined individually based on the subjective detection threshold to single electrical pulses, assessed using the method of limits separately on each forearm (order counterbalanced across participants). Starting from 0.4 mA, the intensity of the electrical stimulus was gradually decreased or increased by steps of 0.01 mA, depending on whether the stimulus was perceived or not, up to several reversals around a stable value that was considered as the absolute detection threshold. HFS trains were delivered at an intensity corresponding to 10 times this detection threshold, slightly adapted in some participants such that single pulses delivered at that intensity were perceived as similar between the two arms (i.e., less than a 10 points difference on a numerical rating scale extending from 0—not felt at all—to 100—maximal pain—, 50 being defined the transition from a non-painful stimulation to a painful one).

#### Assessment of the high-frequency stimulation-induced increase in pinprick sensitivity

Mechanical pinprick stimulation of the volar forearms skin was used to test the successful induction of secondary hyperalgesia. The assessment was performed using two calibrated mechanical pinprick stimulators, one exerting a force of 128 mN and the other of 64 mN. Such stimuli elicit pinprick sensations related to the activation of mechanosensitive nociceptors (see van den Broeke et al., 2015, 2016a,^2020^ for more details). The 64 mN force was chosen based on previous studies showing that a probe with an exerting force of 64 mN captures the strongest and longest-lasting enhancement in pinprick evoked brain potentials after the induction of secondary hyperalgesia when compared to other forces (van den Broeke et al., 2015, 2016a). The 128 mN force was chosen because it had been used in the replicated study (Filbrich et al., 2020).

The stimuli were applied at a pace of about 1 s on the skin of the two forearms within a circular area of 15–20 mm from the center of the HFS treatment area, and never delivered twice onto the same position on the skin. In line with Filbrich et al. (2020), and with previous studies (van den Broeke and Mouraux, 2014; van den Broeke et al., 2016a), the assessment was repeated three different times throughout the whole study: at T0, before applying HFS as a baseline measurement, at T1, immediately after applying HFS and the attentional task, and finally at T2, 20 min after having applied HFS. T1 was included to check whether the attentional task could lead to a change in mechanical sensitivity immediately after the delivery of HFS. However, any change at T1 might also be associated with ongoing sensation given by the HFS itself, and as such not associated to HFS-induced secondary hyperalgesia. The clearest effect of HFS-induced secondary hyperalgesia, as well as a modulation of this effect through spatial attention, is given by any change that occurs from a baseline assessment to several minutes after the delivery of HFS.

At each time point, six pinprick stimuli were applied on each forearm, three for each probe (128 and 64 mN). The order of the arm to start with, as well as the probe to be used first, were counterbalanced across participants, and the same order was kept for the two subsequent time points T1 and T2. Immediately after each stimulus, participants were asked to rate the intensity felt using a numerical scale from 0 (no felt at all) to 100 (maximal pain), with 50 being the transition from a non-painful stimulation to a painful one.

#### Assessment of the spatial extent of the high-frequency stimulation-induced secondary hyperalgesia

To assess the spatial extent of the HFS-induced area of secondary hyperalgesia, the calibrated pinprick stimulus (64 or 128 mN) were applied along the medial-lateral and proximal-distal axis (i.e., two perpendicular lines drawn from the center of HFS application) by steps of 0.5 cm approaching the area on the arms onto which the high frequency stimulation was applied. The stimulation started 7 cm (for the medial-lateral), and 10 cm (for the proximal-distal) away from the center of the area where HFS was applied (e.g., Cayrol et al., 2020). Participants were asked to look away from the arm or to keep their eyes closed while the pinprick stimuli were applied on the arm, and to verbally indicate when they felt a difference in the perception of the pinprick stimulus (e.g., painful, tingling, pinprick, more intense). When a change was perceived, the experimenter marked that point on the arm. The distance of each point from the center of the electrode was measured (in cm) and recorded on a form. For each arm the spatial extent was measured twice, once per pinprick probe (128 and 64 mN). The order of the arm to start with, as well as the probe to be used first, were counterbalanced across participants.

#### Vibrotactile stimulation

Innocuous tactile stimuli were used for the spatial attention task. These stimuli were delivered to both forearms using vibrotactile transducers driven by audio amplifiers (TL-002-14R Haptuators, Tactile Labs Inc., Montreal, Canada). Two types of stimuli were delivered: standard stimuli consisting of a 250 Hz vibration lasting 20 ms, and target stimuli consisting of two succeeding pulses of the same duration and frequency with an inter-pulse interval of 50 ms. The intensity of stimulation was defined individually for each participant, as described in the next section.

### Procedure

The experimental procedure is illustrated at Figure 1B and lasted about 2 h. To avoid experimenter biases and boost interval validity, two experimenters carried out the study (in Filbrich et al., 2020 only one experimenter conducted the study). Experimenter A was blind to participants’ conditions, which means they did not know which arm was the attended one until the end of the procedure. They performed the assessment of mechanical pinprick sensitivity at the different time points and left the lab during the administration of HFS in combination with the attentional task. Experimenter B was always present in the lab and was the person in charge of instructing participants about the experimental procedure and task completion.

Participants were seated in a chair laying their arms on a table facing palms up and resting their chin on a chinrest. The distance between the two arms was about 20 cm. Experimenter A proceeded by cleaning the participants’ volar forearms with ether and alcohol, and successively defined the location to place the HFS electrodes. At this point, the first measurement (T0) for assessing sensitivity to mechanical stimuli was taken.

Next, Experimenter A placed the HFS electrodes on both forearms and assessed the subjective detection threshold to a single electrical pulse, and participants were familiarized with the vibrotactile stimuli.

The magnitude of the vibrotactile stimulation was adjusted for each participant, so that the perceived intensity could be matched between the two arms. The starting intensity was set at 4 mA. When participants could not perceive the vibrotactile stimuli as the same on both arms, the intensity was adjusted by steps of 0.025 mA until the two stimuli would be perceived as the same. Afterward the discrimination task was introduced. The participants were told that during the task they would experience a series of standard single pulse vibrotactile stimuli (*standard stimulus*) delivered simultaneously on both arms, and that they would occasionally receive a target stimulus characterized by a double vibration pulse (*target stimulus)* on one of the two arms. They were instructed to exclusively pay attention to that arm (the *attended arm*), and to report each target stimulus by lifting as fast as possible their foot off a pedal (participants were free to choose which one of the two feet to use to perform the task). They were also explicitly instructed to ignore all stimuli delivered to the other arm (the *unattended arm*). To emphasize the aim of the task, participants were informed that (1) responding too late, (2) responding to standard stimuli, or (3) missing the target stimulus, were considered as mistakes. The speed instruction was solely intended to motivate the participants, reaction times were in fact not recorded. The arm to attend to was counterbalanced across participants so that Experimenter A could not know which arm was the attended one. Depending on the random assigned condition, it was either the arm the least or the most sensitive to a single electrical pulse. To make sure the task was understood properly, participants were presented three times with a sequence of standard stimuli followed by a target stimulus on the attended arm. The task began only if participants were able to detect all the three target stimuli by lifting their foot off the pedal.

During the task, participants, with their chin resting on the chin rest, fixated a dot placed in front of them about 60 cm distant. Environmental noises were masked by white noise delivered through earplugs. A total of 93 standard vibrotactile stimuli were delivered on each arm simultaneously at a random time interval between 2 and 5 s. On the attended arm only, 10 out of 93 were target stimuli. The targets were delivered randomly among the standard ones with the restriction that the minimum time interval between two consecutive targets was 10 s (two targets were therefore never presented one after the other). When a target stimulus was delivered on the attended arm, the unattended arm simultaneously received a standard stimulus. In total, the task lasted 4 min. During the first minute of the task, vibrotactile stimuli were delivered alone, with the occurrence of at least two target stimuli. For the succeeding 2 min, HFS was applied concomitantly to the task. Vibrotactile stimuli were never delivered together with a train of HFS pulses and the minimum time interval between the delivery of an HFS train and a target vibrotactile stimulus was about 2 s. As in Filbrich et al. (2020), when the first train of HFS was going to be delivered, to avoid any impulsive movements consecutive to HFS, Experimenter B gently held the arms of the participant. For the last minute of the task, only vibrotactile stimuli were delivered, with at least the presence of two target stimuli (see Figure 1C for a visual description of the task).

At the end of the task, electrodes and vibrotactile transducers were removed, and Experimenter A came back to assess pinprick sensitivity at T1. Same was done at T2, 20 min later. Additionally, at T2, assessment of the spatial extent of increased pinprick sensitivity was performed.

At the end of the procedure participants were debriefed and compensated for their participation.

### Measures

For each participant, the 3 intensity perception ratings taken for each arm, time point, and pinprick force were averaged before further analyses. For each measurement, we reported median, interquartile range, and value ranges. For an estimate of the spatial extent of increased pinprick sensitivity, the medial-lateral extent was calculated by summing the medial and lateral distances, and the distal-proximal extent was calculated by summing the distal and proximal distances.

### Data analysis

The analyses were performed using RStudio (Version 1.3.1093) for macOS Mozilla/5.0, and were conducted according to our initial pre-registered plan (https://osf.io/84y9j/).

For all analyses, level of significance was considered with a *p*-value < 0.05. If normality assumptions were met, a one-sided paired sample *t*-test was used to compare responses at the attended vs. unattended arm. Effect sizes were calculated using Cohen’s *d* considering a value below 0.3 *negligible*, equal to 0.3 *small*, equal to 0.5 *medium*, and = 0.8 *large*. When assumptions of normality were not met, a non-parametric test was used, specifically a *one-sided paired sample Wilcoxon rank test*. In this case, results were reported using *z scores* and effect sizes were calculated using Pearson’s r coefficient, with *r* of 0.10 considered *small*, 0.30 considered *medium*, and 0.50 considered *large*.

Confirmatory and sensitivity analyses only differed on the data analyzed. Confirmatory analyses were exclusively based on the data collected from the 128 mN pinprick stimuli while sensitivity analyses were based on the data from the 64 mN pinprick stimuli.

### Increased sensitivity to mechanical pinprick stimuli

To check if participants developed an increased sensitivity to mechanical pinprick stimuli consecutive to HFS, intensity of perception ratings measured at T0 were compared to those taken at T1 and T2, respectively using the *Wilcoxon signed-rank test*. This was done separately for each arm.

### Influence of spatial attention on mechanical pinprick sensitivity

To assess whether spatial selective attention modulated the development of mechanical sensitivity to pinprick stimuli, the two arms, attended vs. unattended, were compared in terms of percentage of change in mechanical pinprick sensitivity from time T0 to T1, and from time T0 to T2. The decision to estimate the increase of mechanical sensitivity using the percentage of change in mechanical sensitivity ratings was based on Filbrich et al. (2020) analysis. The percentage of change was computed as: [(mean(T1orT2) –mean(T0))/mean(T0)] *100.

The normality distribution of the percentages of change was tested using a *Shapiro test*, as well as checked visually through Q-Q plots and density curves. Since none of the variables were normally distributed and all positively skewed, we opted for a logarithmic transformation with base 10. Before logarithmic transformation, due to the presence of negative values, we added a positive constant 100, to make all the values positive. According to the *Shapiro test*, after logarithmic transformation, normality assumption was met only for the percentage of change from time T0 to T1 on both arms, but not for the percentage of change from time T0 to T2 for neither of the two arms. On the contrary, all four variables met requirements for normality according to density curves and the Q-Q plots. In the end, in line with our preregistered analysis, a one-sided paired sample *t*-test was used to test primary hypothesis according to which percentage of change would be greater for the attended arm than for the attended one. Yet for a sensitivity check, the primary hypothesis was also tested with percentage of change values not logarithmically transformed using both a non-parametric test, *Wilcoxon signed-rank test*, and a one-sided paired sample *t-test.* All the analysis gave similar results as the ones obtained from the one-sided paired sample *t*-test on logarithmically transformed values (see Supplementary Figures 1, 2 and Supplementary Table 3). As a further sensitivity check, the two arms were compared in terms of mean difference between T2 and T0 and T1 and T0 using a *one- sided paired sample t-tes*t. All the analysis gave similar results as the ones obtained from the comparison between the two arms in terms of percentage of change (see Supplementary Figure 8).

### Influence of spatial attention on the spatial extent of increased pinprick sensitivity

To assess the influence of spatial attention on the spatial extent of increased sensitivity to mechanical pinprick stimuli, we compared the attended and unattended arms separately along the medial-lateral and the proximal-distal axis (Filbrich et al., 2020). Since all variables met normality requirements, the two arms were compared using a *one sided-paired sample t-test*. For each measurement we reported mean and standard deviation (see Supplementary Table 2 for assessment with the probe of 128 mN and Supplementary Table 4 for the 64 mN one).

### Performance on the vibrotactile discrimination task

Initially, as expressed in the preregistration and in line with Filbrich et al. (2020), we planned to include in the final analysis only participants able to detect at least half of the target stimuli presented (i.e., 5 out of 10). However, after data collection, we deviated from this decision, and we analyzed all the participants regardless of the total amount of target stimuli detected. We believe that it cannot be assumed that a good performance (defined in the previous study as the capacity to detect at least half out of the total amount of target stimuli) is an index of having paid attention solely to one of the two arms. The task was only meant to drive attention toward one of the two arms, but its difficulty was not tailored to the participant’s ability to detect the target stimuli. We cannot be sure that the participants that performed the task correctly were the only ones paying attention to the attended arm. It could also be that participants who better performed the task, found it easier and so were more distracted by the painful stimuli delivered on both arms. Similarly, it could also be that participants that found the task more challenging to perform were more prone to stay focused on the task. To this end, to avoid excluding some useful data, we decided to include all the participants regardless of total detected target stimuli. However, for completeness and consistency with the previous study, we also ran the analysis excluding the participants not able to detect at least half of the target stimuli. All the analysis gave similar results (see Supplementary Figures 6, 7).

## Results

### Participants

From the final sample, five out of seventy-two participants were excluded, two for having declared to not have been able to pay attention to the target stimuli, two others for not having been able to handle HFS, and one for lost data. The data of one additional participant was discarded from the analysis aimed to compare the increase in mechanical sensitivity between the two arms, since they declared to not have been consistent with the self-reported ratings from one time point to another. However, they were included when the two arms were compared in terms of spatial extent of the area.

Only for the ratings obtained from the probe with an exerting force of 64 mN, after computing percentage of change, two other participants were excluded since they rated perceived intensity at T0 as equal to 0.

In the end, the data from 67 participants was analyzed [Age = 23.9 ± 3.7 (Mean ± *SD*); 82.1% right-handed; 67.2% female] (see Supplementary Table 1).

The values of the subjective threshold to a single electrical pulse were not significantly different between the right arm (0.28 mA ± 0.08) and left arm (0.26 mA ± 0.08), as confirmed by the paired-sample *t*-test: *t*(66) = 1.88, *d* = –0.230, *p* = 0.06.

The 67 participants kept for the confirmatory and sensitive analyses detected on average 6.32 (± 2.6) out of 10 target stimuli and committed on average 8.9 (± 10.2) false alarms. Out of the 67 participants, 36 were able to detect at least 5 out of 10 target stimuli with less than 10 false alarms.

### Increased sensitivity to mechanical pinprick stimuli

On average HFS induced a significant increase of pinprick sensitivity at both the attended and the unattended arms in response to the 128 mN pinprick stimulus.

**FIGURE 2:**
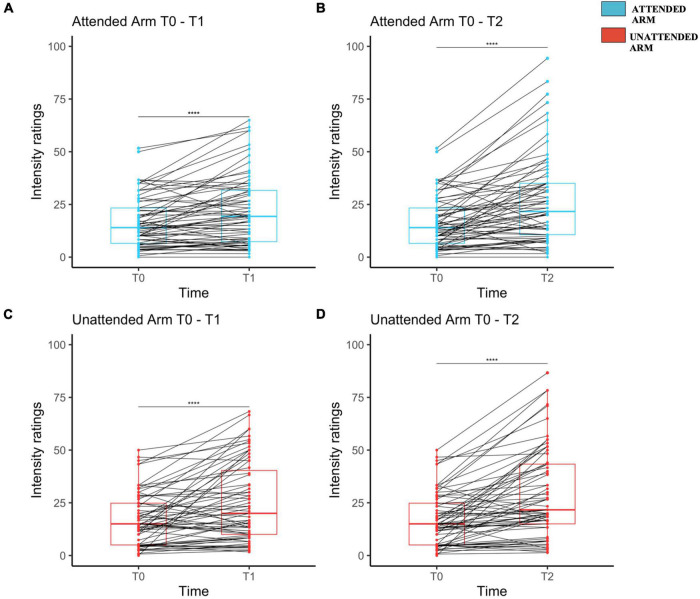
Mechanical pinprick sensitivity measured separately on each arm and for each time point. Attended arm at T1 **(A)** and T2 **(B)** both in comparison to T0. Unattended Arm at T1 **(C)** and T2 **(D)** both in comparison to T0. The red (unattended arm) and the blue (attended arm) bold line within each boxplot represents the median of the intensity ratings at each time point. Median intensity of perception ratings measured at T0 were compared to the one at T1 and T2 though a Wilcoxon signed-rank test, separately for each arm. Each dot represents a single participant perceived intensity on the rating scale at the measured time point for Attended Arm from T0 to T1 **(A)**, and from T0 to T2 **(B)**, as well as for the Unattended Arm from T0 to T1 **(C)** and from T0 to T2 **(D)**. The four stars indicate that there was a significant increase in the perceived intensity.

For the 128 mN stimuli applied on the attended arm, as compared to T0, intensity rating were larger at T1 (T1: Mdn = 19.3, IQR = 24.3, range = 0–65; V = 353, *z* = –425, *r* = 0.52, *p* < 0.001) and at T2 (T2: Mdn = 21.7, IQR = 24.3, range = 0–94.33; *V* = 252, *z* = –5.26, *r* = 0.64, *p* < 0.001) as compared to T0 (T0: Mdn = *14, IQR* = *16.8*, range = 0–51.6) (see Figures 2A,B, respectively). For the same stimuli applied on the unattended arm, as compared to T0, intensity ratings were also larger at T1 (T1: Mdn = 20, IQR = 30.3, Range = 1.66–68.33; *V* = 393.5, *z* = –4.43, *r* = 0.54, *p* < 0.001) and at T2 (T2: Mdn = 21.7, IQR = 28.3, Range = 1.33–86.66; *V* = 234.5, *z* = –5.56, *r* = 0.68, *p* < 0.001) compared to T0 (T0: Mdn = 15, IQR = 19.8, Range = 0–50) (see Figures 2C,D and Supplementary Table 2).

HFS also induced a significant increase of pinprick sensitivity at both attended and unattended arms in response to the 64 mN pinprick stimuli. For the attended arm, as compared to T0 (T0: Mdn = 10, IQR = 11.7, Range = 0–43.33), the intensity ratings were larger at T1 (T1: Mdn = 14, IQR = 20, Range = 0–71.67; *V* = 295, *z* = –4.97, *r* = 0.61, *p* < 0.001) and at T2 (T2: MdN = 18.3, IQR = 24, Range = 1.66–86.66; *V* = 227, *z* = –5.52, *r* = 0.675, *p* < 0.001). For the same stimuli applied on the unattended arm, as compared to T0 (T0: MdN = 10, IQR = 13.5, Range = 0–53), intensity rating were larger at T1 (T1: MdN = 16.67, IQR = 24.8, Range = 1–75; *V* = 361, *z* = –4.42, *r* = 0.53, *p* < 0.001) and at time T2 (T2: MdN = 20, IQR = 30.8, Range = 0.66–86.67; *V* = 204, *z* = –5.41, *r* = 0.66, *p* < 0.001) (see Supplementary Figure 3 and Supplementary Table 4).

### Influence of spatial attention on the sensitivity to mechanical pinprick stimuli

**FIGURE 3:**
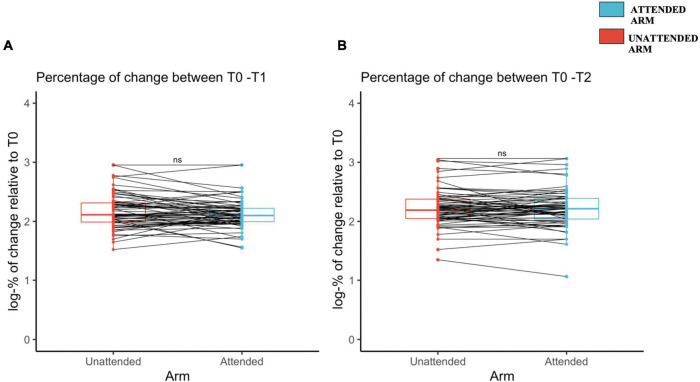
Increased mechanical pinprick sensitivity induced by high-frequency stimulation (HFS) between the unattended and attended arm **(A)** immediately after the induction of high-frequency stimulation, and **(B)** 20 min later, assessed using a probe with an exerting force of 128 mN. The red (unattended arm) and the blue (attended arm) bold lines in the boxplots represent the median of the perceived intensity in terms of percentage of change logarithmically transformed. A one-sided paired sample *t*-test was used to compare the log-transformed percentage of change in mechanical sensitivity from T1 to T0 **(A)** and from T2 to T0 **(B)**. Each dot represents a single participant perceived intensity in terms of percentage of change logarithmically transformed for each arm and at the measured time point, T1 **(A)** and T2 **(B)**. The continuous line indicated with “ns” means that there was no significant difference between the two conditions, attended and unattended.

The percentages of change of mechanical sensitivity, assessed with the 128 mN pinprick stimuli, was not statistically different between the attended and unattended arms, neither at T1 [attended arm: 51.6 ± 116 (2.11 ± 0.23 log-transformed); unattended arm: 83.9 ± 151 (2.16 ± 0.28 log-transformed); *t*(65) = –1.87, *d* = –0.23, *p* = 0.96], nor at T2 [attended arm: 114 ± 198 (2.21 ± 0.32 log-transformed); unattended arm: 117 ± 207 (2.22 ± 0.30 log-transformed); *t*(65) = –0.0302, d = –0.0372, *p* = 0.61] (see Figures 3A,B, respectively and Supplementary Table 2).

The percentage of change assessed with the 64 mN pinprick stimuli was also not statistically different between the attended and unattended arms, neither at T1 [attended arm: 173 ± 121 (2.17 ± 0.24 log-transformed); unattended arm: 192 ± 150 (2.17 ± 0.32 log-transformed); *t*(63) = –0.76, *d* = 0.09, *p* = 0.22], nor at T2 [attended arm: 231 ± 182 (2.26 ± 0.30 log-transformed); unattended arm: 220 ± 164 (2.24 ± 0.30 log-transformed); *t*(63) = 0.17, d = –0.02, *p* = 0.56] (see Supplementary Figures 4A,B, respectively).

### Influence of spatial attention on the spatial extent of increased pinprick

**FIGURE 4:**
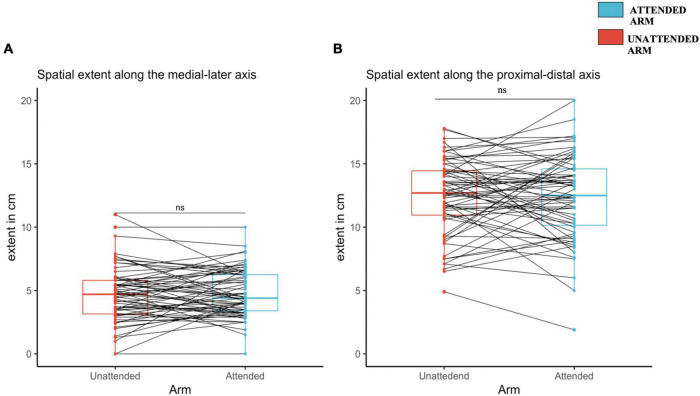
Spatial extent of increased pinprick sensitivity induced by high frequency stimulation (HFS), assessed using a probe with an exerting force of 128 mN at the attended and the unattended arm, 20 min after HFS. **(A)** Medial-later axis. **(B)** Proximal-distal axis. The red (unattended arm) and the blue (attended arm) bold lines in the boxplots represents the median of the extent of the area of increased mechanical sensitivity. A one-sided paired sample *t*-test was used to compare the spatial extent in mechanical sensitivity along the medial-lateral axis **(A)** and the proximal-distal axis **(B)**. Each dot represents a single participants extent of the area of increased sensitivity along the two different axes, **(A)** for medial-lateral, and **(B)** for proximal-distal. The continuous line indicated with “ns” means that there was no significant difference between the two conditions, attended and unattended.

For the 128 mN pinprick stimuli, the length of the medial-lateral axis was not significantly different on the attended arm (4.8 ± 0.1.9) as compared to the unattended arm [4.7 ± 2.2; *t*(66) = 0.57, *d* = 0.069, *p* = 0.28] (see Figure 4A). The same was observed for the proximal-distal axis since there was no statistical difference between the attended arm (12.4 ± 3.4) and the unattended arm [12.4 ± 3; *t*(66) = –0.07, *d* = –0.009, *p* = 0.53) (see Figure 4B).

For the 64 mN pinprick stimuli, the length of the medial-lateral axis was also not statistically different between the attended arm (4.37 ± 1.84) and the unattended arm [4.32 ± 1.88; *t*(66) = 0.26, d = 0.033, *p* = 0.39]. The same was observed for the proximal-distal axis [attended arm: 12.1 ± 3.54; unattended arm: 11.6 ± 2.83; *t*(66) = 1.29, *d* = 0.15, *p* = 0.10] (see Supplementary Figures 5A,B, respectively, and Supplementary Table 4).

## Discussion

We investigated the role of selective spatial attention on the development of secondary hyperalgesia induced experimentally in healthy volunteers. Specifically, we hypothesized that focusing attention on one of the two forearms while high-frequency stimulation was simultaneously applied on both forearms, would induce a greater increase in mechanical pinprick sensitivity on the attended arm as compared to the unattended one. HFS induced a significant increase in pinprick sensitivity at both forearms and at both time points. However, contrary to our expectations, both confirmatory and sensitivity analyses showed that there was no significant difference between the two arms compared in terms of percentage of change in mechanical pinprick sensitivity at T1 and T2 relative to T0 and compared in terms of the spatial extent of secondary hyperalgesia along the medial-lateral and proximal-distal axis. We therefore did not replicate the observations made by Filbrich et al. (2020).

### Top-down vs. bottom-up control of attention

Our results suggest that, at a group level, spatial attention does not selectively affect the development of secondary hyperalgesia, at least when secondary hyperalgesia is induced simultaneously at an attended vs. an unattended body location. It should be stressed that the trains of electrical stimuli constituting HFS generate an intense, painful, and thus highly salient sensation that may interfere with the ability of participants to selectively focus their attention on a single arm. Studies have indeed shown that the ability to selectively focus or discard attention from painful stimuli depends on the balance between bottom-up and top-down factors (Crobez et al., 1994; Eccleston and Crombez, 1999; Legrain et al., 2009a,b, 2011a,2011b; van Ryckeghem et al., 2011). The salience of a stimulus represents its ability to stand out relative to other surrounding stimuli, facilitating the involuntary capture of attention in a bottom-up way. The cognitive load characterizes the effort paid to concentrate on a task. It allows a top-down maintenance of attention to keep focusing on a task while preventing irrelevant stimuli to distract attention. For instance, Legrain et al. (2009b) showed that, when performing a visual task, the sudden and novel occurrence of nociceptive stimuli increases the magnitude of brain responses toward the nociceptive stimuli and distracts participants, thereby slowing their reaction times to task-relevant visual stimuli. However, when the visual task is more difficult and requires more effort, the involuntary capture of attention by the nociceptive stimuli is reduced (Legrain et al., 2013).

In our study, the performance accuracy of the participants was, on average, quite low. Even though they were instructed to ignore HFS and focus exclusively on the target vibrotactile stimuli delivered on one of the two forearms, it could be that the saliency of HFS applied on both arms simultaneously, prevented participants from focusing their attention toward only one of the two forearms. Alternatively, it could be that the tactile discrimination task was not demanding enough to prioritize the use of attentional resources to detect vibratory targets and to prevent attention from being captured by HFS. To increase top-down control over attentional selectiveness and minimize involuntary capture of attention by distractors, Legrain et al. (2009a) proposed that the task should be demanding enough to consume a large part of the attentional resources and prevent them from being scattered and used for task-irrelevant distractions (cognitive load hypothesis). For example, it has been shown that working memory tasks, which generally require more effort to be performed, can inhibit the attentional capture by nociceptive stimuli (e.g., Legrain et al., 2011a,b, 2013) and reduce the induced pain (e.g., Buhle and Wager, 2010; Verhoeven et al., 2011). It was also proposed that the task should hold an attentional set active, i.e., the mental set of stimulus features used as search template to identify stimuli that are goal-relevant, that is restricted to the to-be-attended targets and share as few features as possible with potentially distracting stimuli to prevent distractors from capturing attention (Legrain et al., 2009a). For example, van Ryckeghem et al. (2013) encouraged their participants to focus their attention on an auditory discrimination task to decrease pain in response to electrocutaneous stimuli. They showed that, as compared to a condition during which the task was only defined according to the sensory modality to be attended, when the auditory targets were additionally defined according to a particular spatial location, the reduction in pain was greater for the somatosensory stimuli delivered at another location. In other words, the less the stimuli to be ignored share features with those of the ongoing task, the easier it is to divert attention from them.

It could thus be that in our study, the vibrotactile attentional task was not engaging enough to drive attention exclusively toward one arm and avoid attention to be captured back by the nociceptive stimuli applied to the other arm.

A further suggestion might be that the vibrotactile task acted as a distractor from the electrical HFS pulses, which led to a reduced development of sensitization on both arms. Therefore, the distracting effect would have been independent of the spatial location of the target vibrotactile stimuli. This hypothesis is plausible considering that the increase in mechanical sensitivity from T0 to T2, despite reaching statistical significance, was on average of only ca. 5 points on the NRS intensity scale (i.e., 0–100). This increase was smaller than the one generally found in other studies that used a similar method to induce secondary hyperalgesia (see for instance Klein et al., 2004, 2008; van den Broeke et al., 2016a). Rusheweyh et al. (2011) observed that when participants were engaged in a somatosensory discrimination task, the magnitude of the nociceptive withdrawal reflexes and pain perception were both reduced. If similar unselective aspects of attention also impacted the induction of sensitization during the application of HFS in our present study, this could imply that being involved in a cognitive task unrelated to the induction of sensitization could be enough to minimize it. However, present data cannot support such a hypothesis since we did not use a condition without any concomitant task as a control.

Future experiments aiming to further explore the possible effects of spatial attention on HFS -induced secondary hyperalgesia could use a cognitive task that restricts the attentional set as much as possible to one part of the body and that strongly engages attentional resources to prevent them from being allocated to the other part of the body.

### Perceived utility of attending to a noxious stimulus

According to the adaptive gain theory (Aston-Jones and Cohen, 2005), the locus coeruleus-noradrenergic system (LC-NE) regulates and optimizes behavioral performance by considering the benefits that can be gained by engaging in a task. When a task is associated with high utility, in terms of motivation or perceived reward, it is more likely that a person will engage in the task to profit from it (exploitation). Contrary, when the task utility is low, it is more likely that a person will search elsewhere for utility thus adopting more flexible and distractible behavior (exploration). These two modalities correspond to a different activation of the locus coeruleus, exploitation being associated with a higher phasic activation of the LC, while exploration producing higher tonic activation (Aston-Jones and Cohen, 2005). Highly salient stimuli, such as painful stimuli, are likely to be prioritized due to their intrinsic motivational significance (Aston-Jones and Cohen, 2005). In an animal study, showed that the LC responds physically to a noxious footshock stimulation (Palkovits et al., 1999; Sara and Bouret, 2012), suggesting that when confronted with a salient stimulus, as in this case a noxious electrical stimulation, it is more advantageous to reduce exploration and prioritize the painful stimulus. Recently, van den Broeke et al. (2019), using pupil size as an index of LC activity, showed that following HFS, mechanical pinprick stimulation produced a stronger phasic pupil dilation, indicating a stronger phasic activation of the LC.

In our study, participants were placed in a context producing two alternatives: focus attention toward the vibrotactile stimuli with the aim of performing the instructed task, or prioritize the painful electrical stimuli delivered on both arms. Pertinent to *the adaptive gain theory*, it could be that, due to the intensity and salience of HFS, participants recognized prioritization of the painful stimuli as having a greater perceived utility.

Future studies attempting to investigate the interactions between selective attention and the development of secondary hyperalgesia could attempt to bias prioritization toward non-painful target stimuli by boosting engagement through positive incentives. This could motivate participants to allocate their attention to the task-relevant stimuli, rather than the nociceptive stimuli (Verhoeven et al., 2010).

### Conclusion

Our results show that in a condition of bilateral application of HFS at two separate body sites, the left and right volar forearms, a task aiming at focusing spatial attention toward one of the two forearms does not increase the magnitude of mechanical pinprick sensitivity on the attended arm as compared to the unattended arm.

## Data availability statement

The original contributions presented in the study are publicly available. This data can be found here: https://osf.io/84y9j/.

## Ethics statement

The studies involving human participants were reviewed and approved by Commission d’Ethique Biomédicale Hospital-Facultaire, Saint-Luc University Hospital & UCLouvain. The patients/participants provided their written informed consent to participate in this study.

## Author contributions

DDP contributed to conception, design and methodology of the study, collected the data, performed the statistical analysis, wrote the original draft, revised, and edited the manuscript. VL acquired funding, supervised the project, contributed to conception, design and methodology of the study, revised, and edited the manuscript. M-LV and AK contributed to collect the data, revised, and edited the manuscript. LF and AM supervised the project, revised, and edited the manuscript. All authors contributed to manuscript revision, read, and approved the submitted version.

## References

[B1] Aston-Jones G. Cohen J. D. (2005). An integrative theory of locus coeruleus-norepinephrine function: Adaptive gain and optimal performance. *Annu. Rev. Neurosci.* 28 403–450. 10.1146/annurev.neuro.28.061604.135709 16022602

[B2] Buhle J. Wager T. D. (2010). Performance-dependent inhibition of pain by an executive working memory task. *Pain* 149 19–26. 10.1016/j.pain.2009.10.027 20129735 PMC4229048

[B3] Button K. S. Ioannidis J. P. A. Mokrysz C. Nosek B. A. Flint J. Robinson E. S. J. (2013). Power failure: Why small sample size undermines the reliability of neuroscience. *Nat. Rev. Neurosci.* 14 365–376. 10.1038/nrn3475 23571845

[B4] Cayrol T. Lebleu J. Mouraux A. Roussel N. Pitance L. van den Broeke E. N. (2020). Within- and between-session reliability of secondary hyperalgesia induced by electrical high-frequency stimulation. *Eur. J. Pain* 24 1585–1597. 10.1002/ejp.1613 32501583

[B5] Crobez G. Baeyens F. Eelen P. (1994). Sensory and temporal information about impending pain: The influence of predictability on pain. *Behav. Res. Ther.* 32 611–622. 10.1016/0005-7967(94)90015-9 8085989

[B6] Crombez G. Eccleston C. Van den Broeck A. Goubert L. Van Houdenhove B. (2004). Hypervigilance to pain in fibromyalgia: The mediating role of pain intensity and catastrophic thinking about pain. *Clin. J. Pain* 20 98–102. 10.1097/00002508-200403000-00006 14770049

[B7] Crombez G. van Damme S. Eccleston C. (2005). Hypervigilance to pain: An experimental and clinical analysis. *Pain* 116 4–7. 10.1016/j.pain.2005.03.035 15927387

[B8] Eccleston C. Crombez G. (1999). Pain demands attention: A cognitive-affective model of the interruptive function of pain. *Psychol. Bull.* 125 356–366. 10.1037/0033-2909.125.3.356 10349356

[B9] Eippert F. Finsterbusch J. Bingel U. Büchel C. (2009). Direct evidence for spinal cord involvement in placebo analgesia. *Science* 326 404–404. 10.1126/science.1180142 19833962

[B10] Filbrich L. van den Broeke E. N. Legrain V. Mouraux A. (2020). The focus of spatial attention during the induction of central sensitization can modulate the subsequent development of secondary hyperalgesia. *Cortex* 124 193–203. 10.1016/j.cortex.2019.11.014 31901709

[B11] Gousset S. Mouraux A. Van Den Broeke E. N. (2020). Burst-like conditioning electrical stimulation is more efficacious than continuous stimulation for inducing secondary hyperalgesia in humans. *J. Neurophysiol.* 123 323–328. 10.1152/jn.00675.2019 31825708 PMC6985853

[B12] Harte S. E. Harris R. E. Clauw D. J. (2018). The neurobiology of central sensitization. *J. Appl. Behav. Res.* 23:12137. 10.1111/jabr.12137

[B13] Ioannidis J. P. (2005). Why most published research findings are false. *PLoS Med.* 2:e124. 10.1371/journal.pmed.0020124 16060722 PMC1182327

[B14] Klede M. Handwerker H. O. Schmelz M. (2003). Central origin of secondary mechanical hyperalgesia. *J. Neurophysiol.* 90 353–359. 10.1152/jn.01136.2002 12843313

[B15] Klein T. Magerl W. Hopf H. C. Sandkühler J. Treede R. D. (2004). Perceptual correlates of nociceptive long-term potentiation and long-term depression in humans. *J. Neurosci.* 24 964–971. 10.1523/JNEUROSCI.1222-03.2004 14749441 PMC6729815

[B16] Klein T. Stahn S. Magerl W. Treede R. D. (2008). The role of heterosynaptic facilitation in long-term potentiation (LTP) of human pain sensation. *Pain* 139 507–519. 10.1016/j.pain.2008.06.001 18602755

[B17] LaMotte R. H. Shain C. N. Simone D. A. Tsai E. F. (1991). Neurogenic hyperalgesia: Psychophysical studies of underlying mechanisms. *J. Neurophysiol.* 66 190–211. 10.1152/jn.1991.66.1.190 1919666

[B18] Latremoliere A. Woolf C. J. (2009). Central sensitization: A generator of pain hypersensitivity by central neural plasticity. *J. Pain* 10 895–926. 10.1016/j.jpain.2009.06.012 19712899 PMC2750819

[B19] Legrain V. Crombez G. Mouraux A. (2011a). Controlling attention to nociceptive stimuli with working memory. *PLoS One* 6:e20926. 10.1371/journal.pone.0020926 21687745 PMC3110248

[B20] Legrain V. Crombez G. Plaghki L. Mouraux A. (2013). Shielding cognition from nociception with working memory. *Cortex* 49 1922–1934. 10.1016/j.cortex.2012.08.014 23026759

[B21] Legrain V. Crombez G. Verhoeven K. Mouraux A. (2011b). The role of working memory in the attentional control of pain. *Pain* 152 453–459. 10.1016/j.pain.2010.11.024 21238855

[B22] Legrain V. Damme S. van Eccleston C. Davis K. D. Seminowicz D. A. Crombez G. (2009a). A neurocognitive model of attention to pain: Behavioral and neuroimaging evidence. *Pain* 144 230–232. 10.1016/j.pain.2009.03.020 19376654

[B23] Legrain V. Perchet C. García-Larrea L. (2009b). Involuntary orienting of attention to nociceptive events: Neural and behavioral signatures. *J. Neurophysiol.* 102 2423–2434. 10.1152/jn.00372.2009 19692512

[B24] Legrain V. Mancini F. Sambo C. F. Torta D. M. Ronga I. Valentini E. (2012). Cognitive aspects of nociception and pain: Bridging neurophysiology with cognitive psychology. *Neurophysiol. Clin.* 42 325–336. 10.1016/j.neucli.2012.06.003 23040703

[B25] Matre D. Casey K. L. Knardahl S. (2006). Placebo-induced changes in spinal cord pain processing. *J. Neurosci.* 26 559–563. 10.1523/JNEUROSCI.4218-05.2006 16407554 PMC6674401

[B26] Navratilova E. Porreca F. (2014). Reward and motivation in pain and pain relief. *Nat. Neurosci.* 17 1304–1312. 10.1038/nn.3811 25254980 PMC4301417

[B27] Palkovits M. Baffi J. S. Pacak K. (1999). The role of ascending neuronal pathways in stress-induced release of noradrenaline in the hypothalamic paraventricular nucleus of rats. *J. Neuroendocrinol.* 11, 529–539. 10444310 10.1046/j.1365-2826.1999.00365.x

[B28] Pfau D. B. Klein T. Putzer D. Pogatzki-Zahn E. M. Treede R.-D. Magerl W. (2011). Analysis of hyperalgesia time courses in humans after painful electrical high-frequency stimulation identifies a possible transition from early to late LTP-like pain plasticity. *Pain* 152 1532–1539. 10.1016/j.pain.2011.02.037 21440369

[B29] Raja S. N. Campbell J. N. Meyer R. A. (1984). Evidence for different mechanisms of primary and secondary hyperalgesia following heat injury to the glabrous skin. *Brain* 107 1179–1188. 10.1093/brain/107.4.1179 6509313

[B30] Rusheweyh R. Kreusch A. Albers C. Sommer J. Marziniak M. (2011). The effect of distraction strategies on pain perception and the nociceptive flexor reflex (RIII reflex). *Pain* 152 2662–2671. 10.1016/j.pain.2011.08.016 21925793

[B31] Salomons T. V. Moayedi M. Erpelding N. Davis K. D. (2014). A brief cognitive-behavioural intervention for pain reduces secondary hyperalgesia. *Pain* 155 1446–1452. 10.1016/j.pain.2014.02.012 24569149

[B32] Sara S. J. Bouret S. (2012). Orienting and reorienting: The locus coeruleus mediates cognition through arousal. *Neuron* 76 130–141. 10.1016/j.neuron.2012.09.011 23040811

[B33] Sprenger C. Eippert F. Finsterbusch J. Bingel U. Rose M. Büchel C. (2012). Attention modulates spinal cord responses to pain. *Curr. Biol.* 22 1019–1022. 10.1016/j.cub.2012.04.006 22608507

[B34] Torta D. M. de Laurentis M. Eichin K. N. von Leupoldt A. van den Broeke E. N. Vlaeyen J. W. S. (2020). A highly cognitive demanding working memory task may prevent the development of nociceptive hypersensitivity. *Pain* 161 1459–1469. 10.1097/j.pain.0000000000001841 32102023

[B35] Tracey I. Mantyh P. W. (2007). The cerebral signature for pain perception and its modulation. *Neuron* 55 377–391. 10.1016/j.neuron.2007.07.012 17678852

[B36] van Damme S. Crombez G. Eccleston C. (2008). Coping with pain: A motivational perspective. *Pain* 139 1–4. 10.1016/j.pain.2008.07.022 18755548

[B37] van Damme S. Legrain V. Vogt J. Crombez G. (2010). Keeping pain in mind: A motivational account of attention to pain. *Neurosci. Biobehav. Rev.* 34 204–213. 10.1016/j.neubiorev.2009.01.005 19896002

[B38] van den Broeke E. N. de Hemptinne P. Mercken M. Torta D. M. Lambert J. Mouraux A. (2020). Central sensitization of nociceptive pathways demonstrated by robot-controlled pinprick-evoked brain potentials. *Clin. Neurophysiol.* 131 2491–2498. 10.1016/J.CLINPH.2020.06.020 32709556

[B39] van den Broeke E. N. Geene N. van Rijn C. M. Wilder-Smith O. H. G. Oosterman J. (2014). Negative expectations facilitate mechanical hyperalgesia after high-frequency electrical stimulation of human skin. *Eur. J. Pain* 18 86–91. 10.1002/j.1532-2149.2013.00342.x 23754275

[B41] van den Broeke E. N. Hartgerink D. M. Butler J. Lambert J. Mouraux A. (2019). Central sensitization increases the pupil dilation elicited by mechanical pinprick stimulation. *J. Neurophysiol.* 121 1621–1632. 10.1152/jn.00816.2018 30785805

[B42] van den Broeke E. N. Lambert J. Huang G. Mouraux A. (2016a). Central sensitization of mechanical nociceptive pathways is associated with a long-lasting increase of pinprick-evoked brain potentials. *Front. Hum. Neurosci.* 10:531. 10.3389/fnhum.2016.00531 27812331 PMC5071355

[B43] van den broeke E. N. Lenoir C. Mouraux A. (2016b). Secondary hyperalgesia is mediated by heat-insensitive afibre nociceptors. *J. Physiol.* 594 6767–6776. 10.1113/JP272599 27377467 PMC5108905

[B44] van den Broeke E. N. Mouraux A. (2014). High-frequency electrical stimulation of the human skin induces heterotopical mechanical hyperalgesia, heat hyperalgesia, and enhanced responses to nonnociceptive vibrotactile input. *J. Neurophysiol*. 111, 1564–1573. 10.1152/jn.00651.2013 24453277

[B45] van den Broeke E. N. Mouraux A. Groneberg A. H. Pfau D. B. Treede R. D. Klein T. (2015). Characterizing pinprick-evoked brain potentials before and after experimentally induced secondary hyperalgesia. *J. Neurophysiol.* 114 2672–2681. 10.1152/jn.00444.2015 26334010 PMC4644227

[B46] van den Broeke E. N. van Rijn C. M. Biurrun Manresa J. A. Andersen O. K. Arendt-Nielsen L. Wilder-Smith O. H. (2010). Neurophysiological correlates of nociceptive heterosynaptic long-term potentiation in humans. *J. Neurophysiol.* 103, 2107–2113. 20164395 10.1152/jn.00979.2009

[B47] Van Ryckeghem D. M. L. Crombez G. Eccleston C. Legrain V. Van Damme S. (2013). Keeping pain out of your mind: The role of attentional set in pain. *Eur. J. Pain* 17, 402–411. 23070963 10.1002/j.1532-2149.2012.00195.x

[B48] van Ryckeghem D. M. L. van Damme S. Crombez G. Eccleston C. Verhoeven K. Legrain V. (2011). The role of spatial attention in attentional control over pain: An experimental investigation. *Exp. Brain Res.* 208 269–275. 10.1007/s00221-010-2477-y 21063689

[B49] van Ryckeghem D. M. van Damme S. Eccleston C. Crombez G. (2018). The efficacy of attentional distraction and sensory monitoring in chronic pain patients: A meta-analysis. *Clin. Psychol. Rev.* 59 16–29. 10.1016/j.cpr.2017.10.008 29126746

[B50] Verhoeven K. Christopher E. Dimitri M. V. R. Valéry L. Geert C. (2011). Distraction from pain and executive functioning: An experimental investigation of the role of inhibition, task switching and working memory. *Eur. J. Pain* 15 866–873. 10.1016/j.ejpain.2011.01.009 21397536

[B51] Verhoeven K. Crombez G. Eccleston C. van Ryckeghem D. M. L. Morley S. van Damme S. (2010). The role of motivation in distracting attention away from pain: An experimental study. *Pain* 149 229–234. 10.1016/j.pain.2010.01.019 20188469

[B52] Woolf C. J. (1983). Evidence for a central component of post-injury pain hypersensitivity. *Nature* 306 686–688. 10.1038/306686a0 6656869

[B53] Woolf C. J. (2011). Central sensitization: Implications for the diagnosis and treatment of pain. *Pain* 152 (Suppl. 3) S2–S15. 10.1016/j.pain.2010.09.030 20961685 PMC3268359

[B54] Woolf C. J. Salter M. W. (2000). Neuronal plasticity: Increasing the gain in pain. *Science* 288 1765–1768. 10846153 10.1126/science.288.5472.1765

