# Supplementary material for: No evidence for an effect of selective spatial attention on the development of secondary hyperalgesia: A replication study
Source: Front Hum Neurosci. 2022 Nov 3;16:997230. doi: 10.3389/fnhum.2022.997230 (PMC9670179; doi:10.3389/fnhum.2022.997230)
Supplement: Supplementary file 1 [file Data_Sheet_1.docx]

**Supplemental Digital Content (SDC)**

**Supplementary Table 1.** **Descriptive statistics of the sample**

| *Measures* | *Values* | *Total (N)* | *Missing(N)* |
| --- | --- | --- | --- |
| **Age (Mean ± Sd)**  **Age (min < med < max)** | 23.9 ± 3.7  18 < 23 < 35 | 67 | 0 |
| **Nationality (N, %)**  1. Belgian  2. French  3. Italian  4. Moroccan  5. Cameroonians  6. Canadian  7. Chinese  8. Croatian  9. Gabonese  10. Indian  11. Swiss  12. Romanian  13. Venezuelan  14. Polish  15. Slovak  16. Spanish & Belgian  17. Moroccan & Belgian | 33,49.3%  10, 14.9 %  7, 10.4 %  3, 4.50 %  2, 3.50 %  1, 1.45 %  1, 1.45 %  1, 1.45 %  1, 1.45 %  1, 1.45 %  1, 1.45 %  1, 1.45 %  1, 1.45 %  1, 1.45 %  1, 1.45 %  1, 1.45 %  1, 1.45 % | 67 | 0 |
| **Gender (N, %)**  Female  Male | 45, 67.2 %  22, 32.8 % | 67 | 0 |
| **Handiness (N, %)**  Right  Left | 55, 82.1 %  12, 17.9 % | 67 | 0 |
| **Baseline pain (Mean ± Sd)** | 1.5 ± 4.9 | 67 | 0 |
| **No Caffeinated beverage 24 hours before the experiment (N, %)** | 57, 85.1% | 67 | 0 |
| **Target stimuli detected or hits**  **(Mean ± Sd)**  **(min < med < max)** | 6.36 ± 2.61  1 < 7 < 10 | 66 | 1 |

| **Left Arm Threshold to a single electrical pulse** | *0.26* ± *0.08 mA*  *0.05 < 0.25 < 0.47* | *67* | *0* |
| --- | --- | --- | --- |
| **Right Arm Threshold to a single electrical pulse** | *0.28* ± *0.08 mA*  *0.12 < 0.28< 0.45* | *67* | *0* |
| **Ratings Right** | *40.24* ± *18.86*  *5.50 < 40.0 < 82.5* | *60* | *7 ^1^* |
| **Ratings Left** | 40.21 ± 19.10  4.50 < 40.5 < 85.0 | 60 | 7 ^1^ |

1. We asked all the participants tested to rate the intensity of 10 times the value of a single electrical pulse separately for each arm to make sure the intensity was perceived as the same on both arms. Due to a mistake, we did not report on the experimenter's form the ratings from 7 out of 67 participants.

**Supplementary Table 2. Mechanical pinprick sensitivity assessed on attended and unattended arm using a probe exerting a force of 128mN in terms of intensity and spatial extent along proximal- distal and medial lateral axis**

| Conditions  Measures | Attend Arm  N = 67 | Unattended Arm  N = 67 |
| --- | --- | --- |
| T0, before High Frequency Stimulation^1^  Mean ± Sd, SE  min < med < max | 16.49 ± 12.07, 1.47  0 < 14.00 < 51.67 | 16.99 ± 12.51, 1.52  0 < 15.00 < 50.00 |
| T1, immediately after High Frequency Stimulation^1^  Mean ± Sd, SE  min < med < max | 21.89 ± 16.71, 2.04  0 < 19.33 < 65.00 | 25.60 ± 18.74, 2.29  1.67 < 20.00 < 68.33 |
| T2, 20 minutes after High Frequency Stimulation^1^  Mean ± Sd, SE  min < med < max) | 27.26 ± 21.16, 2.58  0 < 21.67< 94.33 | 29.11 ± 21.36, 2.60  1.33 < 21.67 < 86.67 |
| Medial -later Axis  Mean ± Sd, SE  min < med < max | 4.8 ± .1.9, .22  0 < 4.4 < 10 | 4.7 ± 2.2, .26  0 < 4.7 < 11 |
| Proximal-distal axis  Mean ± Sd, SE  min < med < max | 12.4 ± 3.4, .41  1.9 < 12.5 < 20 | 12.4 ± 3, .36  4.9 < 12.7 < 17.8 |

1. For both conditions at each time point, the range on the rating scale was the following: 0 “not felt at all”, 50 “transition from intensive sensation to a low painful one”, and 100 “the worst painful sensation imaginable”.

**Supplementary Table 3. Mechanical pinprick sensitivity assessed on attended and unattended arm using a probe with an exerting force of 128mN and expressed in terms of percentage of change from time T0 to T1 and T2**

| Conditions  Measures^1^ | Attend Arm  N = 66 | Unattended Arm  N = 66 |
| --- | --- | --- |
| Percentage of change from T0 to T2 ^1^  Mean ± Sd, SE  min < med < max | 2.21 ± .32, .04  1.1 < 2.2 < 3.1 | 2.22 ± .30, .03  1.3 < 2.2 < 3 |
| Percentage of change from T0 to T1 ^1^  Mean ± Sd, SE  min < med < max | 2.11 ± .23,.02  1.6 < 2.1 < 3 | 2.16 ± .28, .03  1.5 < 2.1 < 3 |
| Percentage of change from T2 to T0  Mean ± Sd, SE  min < med < max | 114 ± 198, 24.3  -88.4 < 63.6 < 1050 | 117 ± 207, 25.4  -77.7 < 55 < 1000 |
| Percentage of change from T1 to T0  Mean ± Sd, SE  min < med < max | 51.6 ± 116, 14.2  -64.5 < 25.6 < 800 | 83.9 ± 151, 18.5  -66.7 < 29.25 < 800 |
|  |  |  |

1. These values refer to the percentage of change after logarithmic transformation


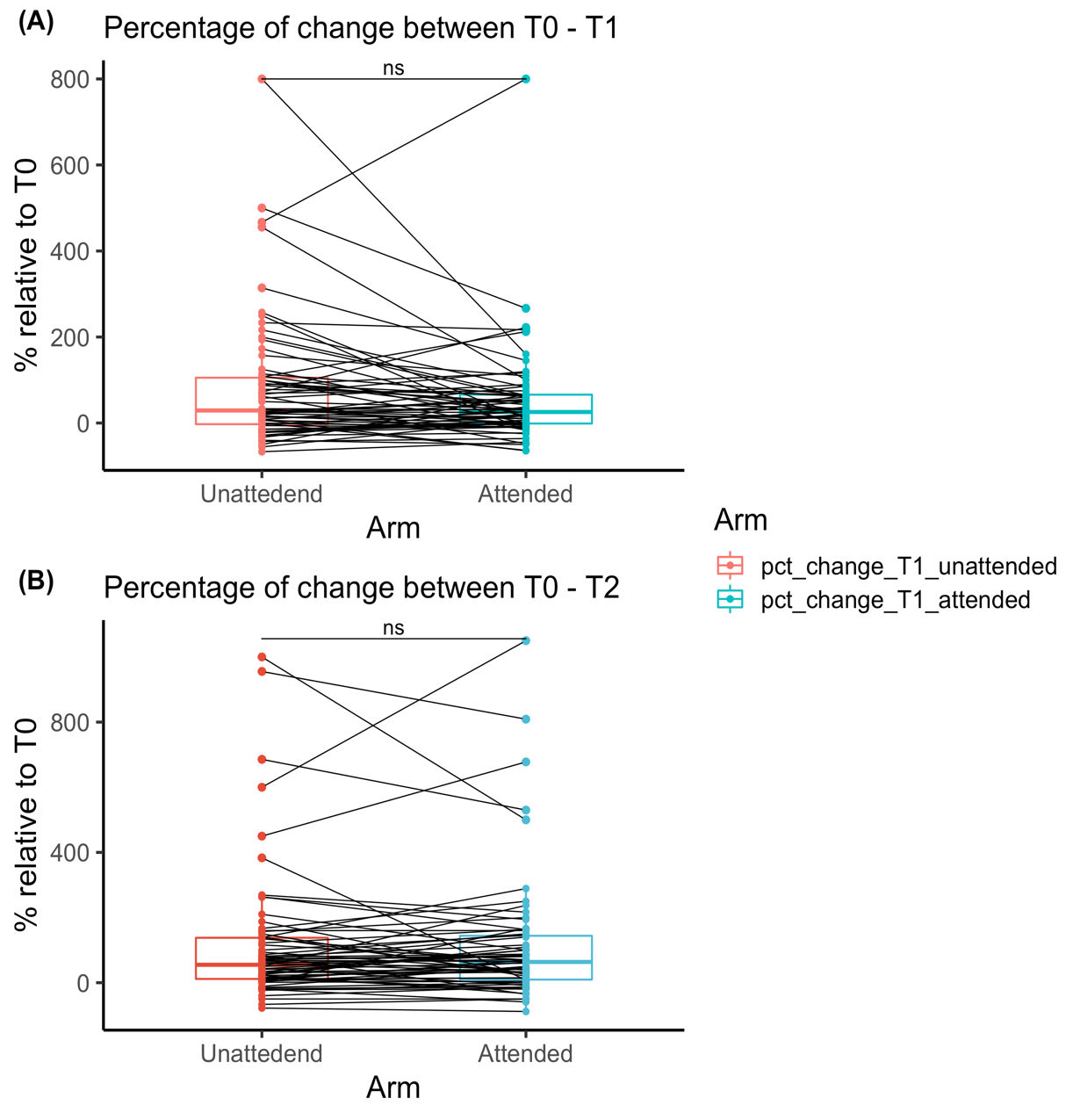


Unattended

Attended


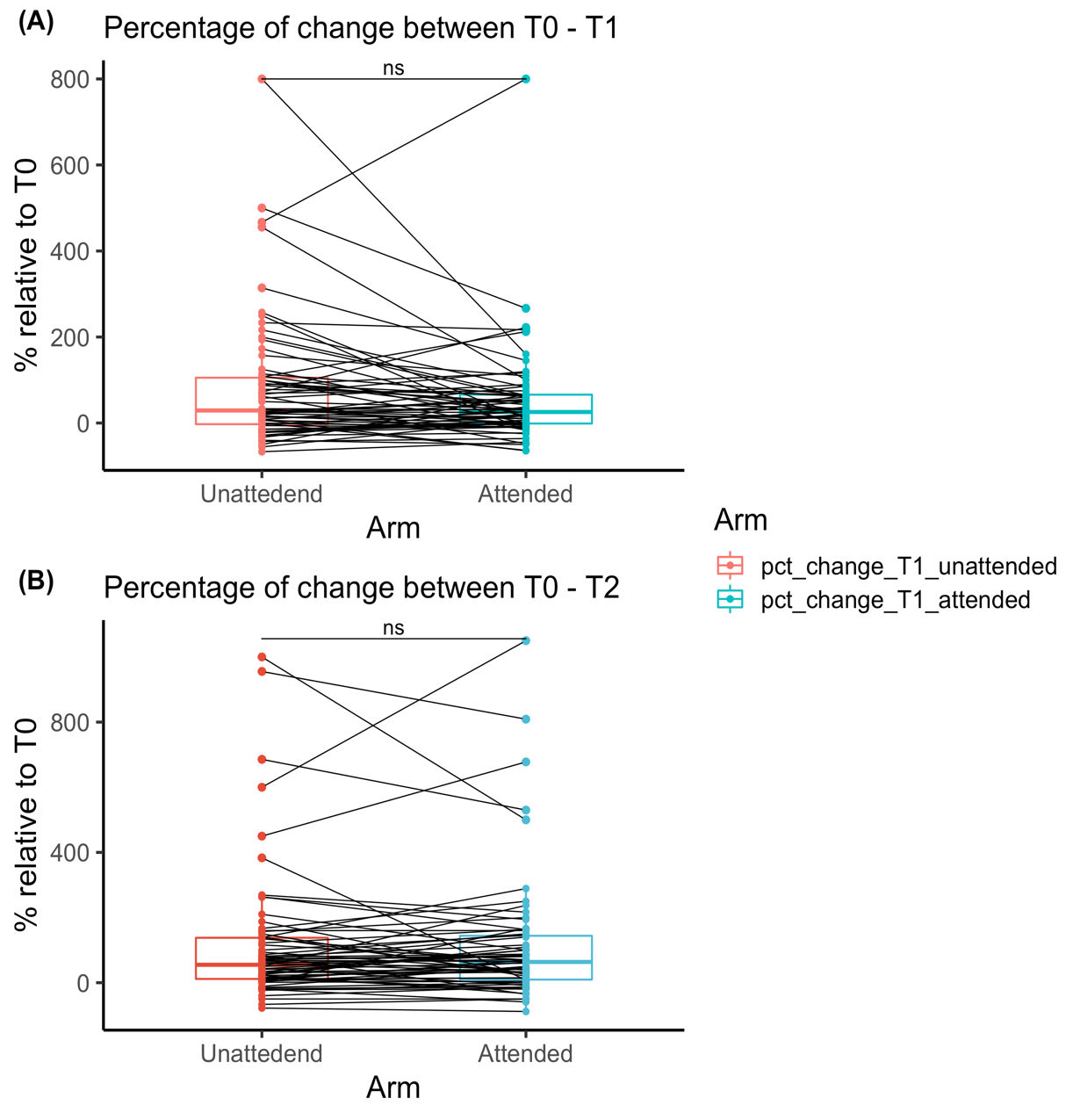


Unattended

Attended

**Supplementary Figure 1 | Sensitivity to mechanical pinprick stimuli in terms of percentage of change comparing attended vs unattended arm using a *one-sided Wilcoxon rank test*. (A) Immediately after the induction of high-frequency stimulation, T1, and (B) 20 minutes later T2.**

The red and the blue bold lines in the boxplots represent the median of the perceived intensity in terms of percentage of change. The red one for the unattended arm and the blue one for the attended arm. Each dot represents a single participants perceived intensity in terms of percentage of change from time T0 to T1 (A), and from time T0 to T2 (B).

The continuous line indicated with “ns” means that the increased sensitivity on the attend arm was not significantly greater than the one developed on the unattended arm. As confirmed by one a sided Wilcoxon rank test: (A) = V = 816,

z = -.058, r = .204, p = .95; (B) V= 1057, z =-.33, r = .05, p = .37.

|  |  |  |
| --- | --- | --- |

Unattended

Attended

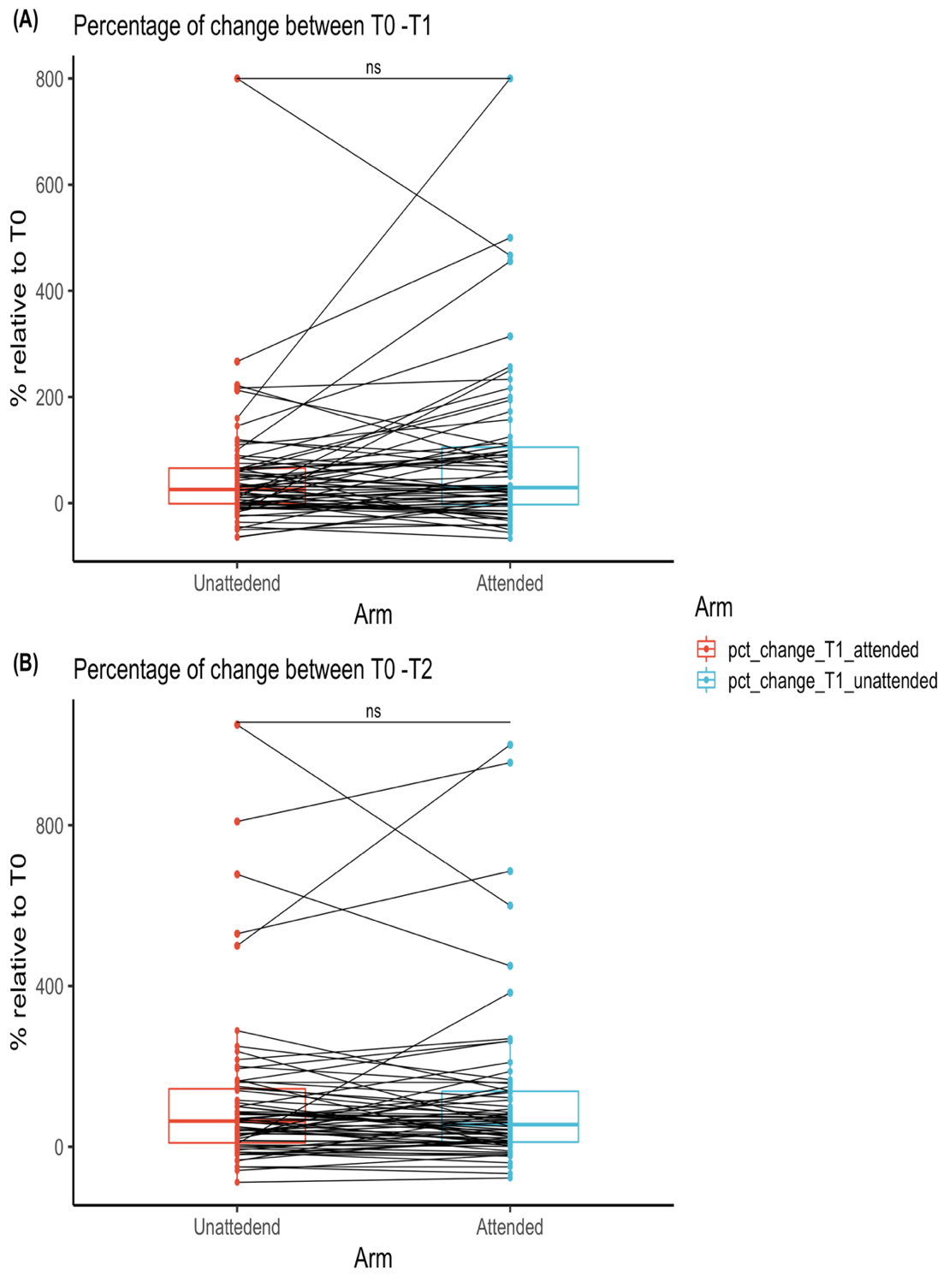


Unattended

Attended

**Supplementary Figure 2|** **Sensitivity to mechanical pinprick stimuli with a force of 128mN in terms of percentage of change comparing attended vs unattended arm using a *one-sided paired sample t test.* (A) Immediately after the induction of high-frequency stimulation, T1, and (B) 20 minutes later T2.**

The red and the blue bold lines in the boxplots represent the median of the perceived intensity in terms of percentage of change. The red one for the unattended arm and the blue one for the attended arm. Each dot represents a single participants perceived intensity in terms of percentage of change from time T0 to T1 (A), and from time T0 to T2 (B).

The continuous line indicated with “ns” means that the increased sensitivity on the attend arm was not significantly greater than the one developed on the unattended arm. As confirmed by the one-sided paired sample t- test (A) = t (65) = -2.09, d = -.25, p= .98; (B) = t (65) = -.228, d= -.028, p=.95.


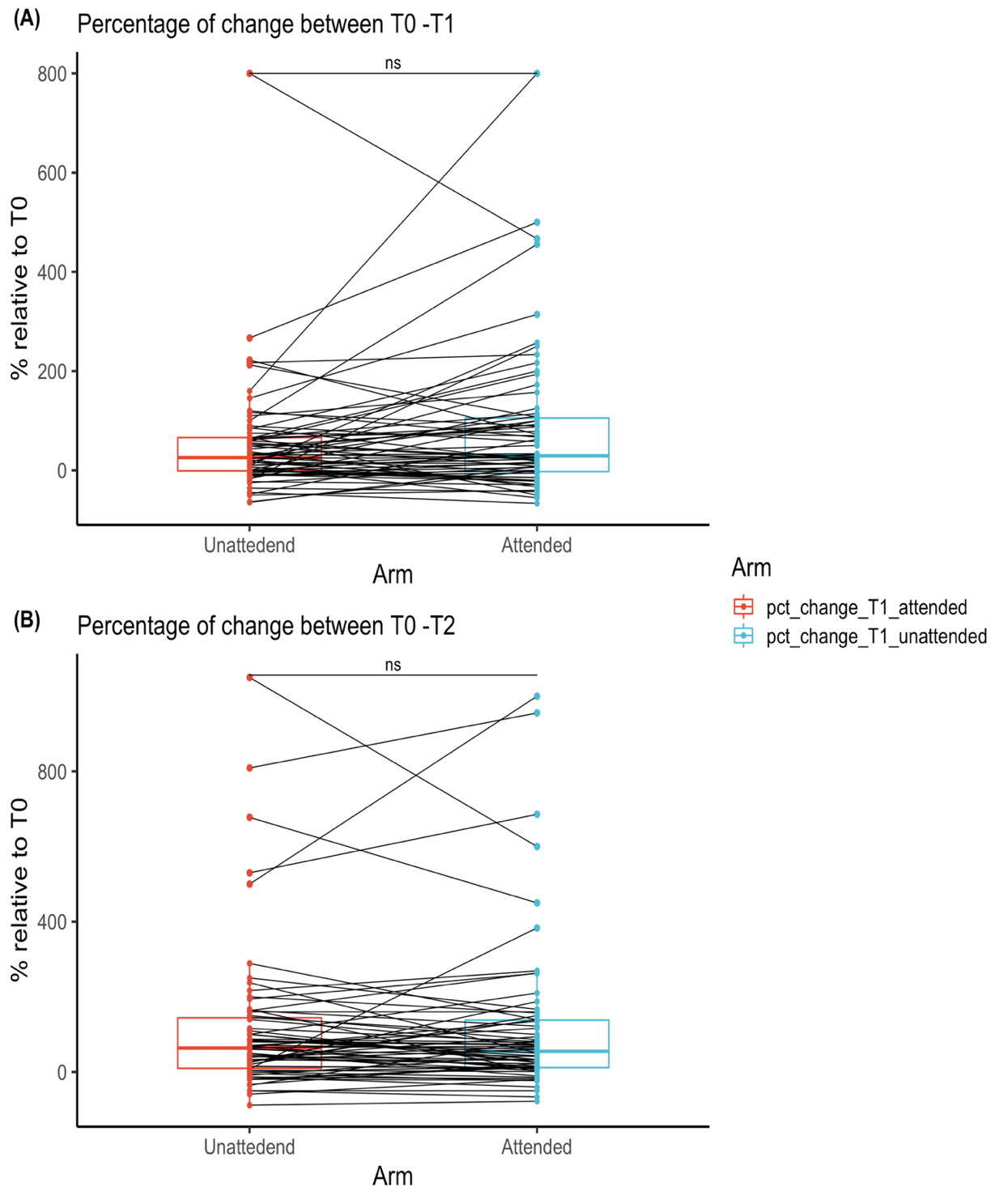


Unattended

Attended

**Supplementary Table 4. Mechanical pinprick sensitivity assessed using a probe exerting a force of 64mN, in terms of intensity and spatial extent along proximal- distal and medial lateral axis**

**Descriptive statistics containing (1) mean, standard deviation and standard error, (2) minimum, median and maximal value for the intensity ratings on each time point and for both conditions**

| Conditions  Measures | Attend Arm  N = 67 | Unattended Arm  N = 67 |
| --- | --- | --- |
| T0, before High Frequency Stimulation^1^  Mean ± Sd, SE  min < med < max | 12.83 ± 10.09, 1.23  0 < 10.00 < 43.33 | 13.40 ± 10.29, 1.25  0 < 10.00 < 53.00 |
| T1, immediately after High Frequency Stimulation^1^  Mean ± Sd, SE  min < med < max | 19.42 ± 16.22, 1.98  0 < 14.00 < 71.67 | 22.75 ± 18.54, 2.26  1 < 16.67 < 75.00 |
| T2, 20 minutes after High Frequency Stimulation^1^  Mean ± Sd, SE  min < med < max) | 25.18 ± 20.70, 2.52  1.66 < 18.33 < 86.66 | 26.18 ± 21.02, 2.56  0.66 < 20.00 < 86.67 |
| Medial-later Axis  Mean ± Sd, SE  min < med < max  Proximal-Distal Axis  Mean ± Sd, SE  min < med < max | 4.4 ± .1.8,.22  0 < 4.2 < 9  12.1 ± 3.5,.43  3.5 < 12.9 < 19.2 | 4.3 ± 1.9, .23  0 < 4.4 < 8.8  11.6 ± 2.8, .34  3.4 < 12.1 < 17 |

1. The range on the rating scale was the following: 0 “not felt at all”, 50 “transition from intensive sensation to a low painful one”, and 100 “the worst painful sensation imaginable”.

 **
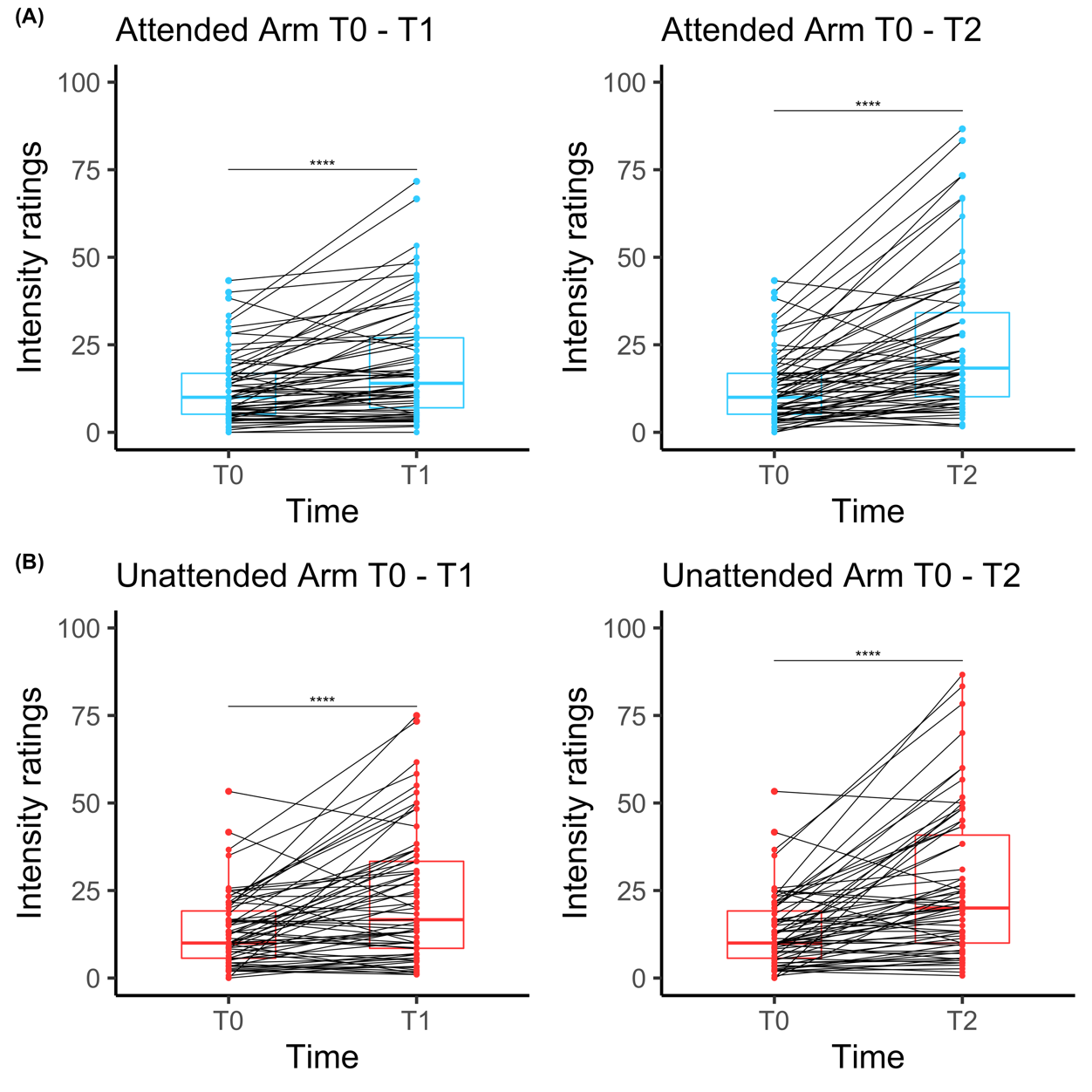
**

**Supplementary Figure 3 | Mechanical pinprick sensitivity measured separately on each arm and for each time point using a probe with an exerting force of 64mN.**

**(A)** Mechanical sensitivity assessed on the attended arm at T1 and T2 in comparison to T0, **(B)** Mechanical sensitivity assessed on the unattended Arm at T1 and T2 both in comparison to T0.

The bold line within each boxplot represents the median of the intensity ratings at each time point. Each dot represents a single participant perceived intensity on the rating scale at the measured time point for both (A) Attended Arm, and (B) Unattended Arm.

The four stars indicate that there was a significant increase in perceived intensity.


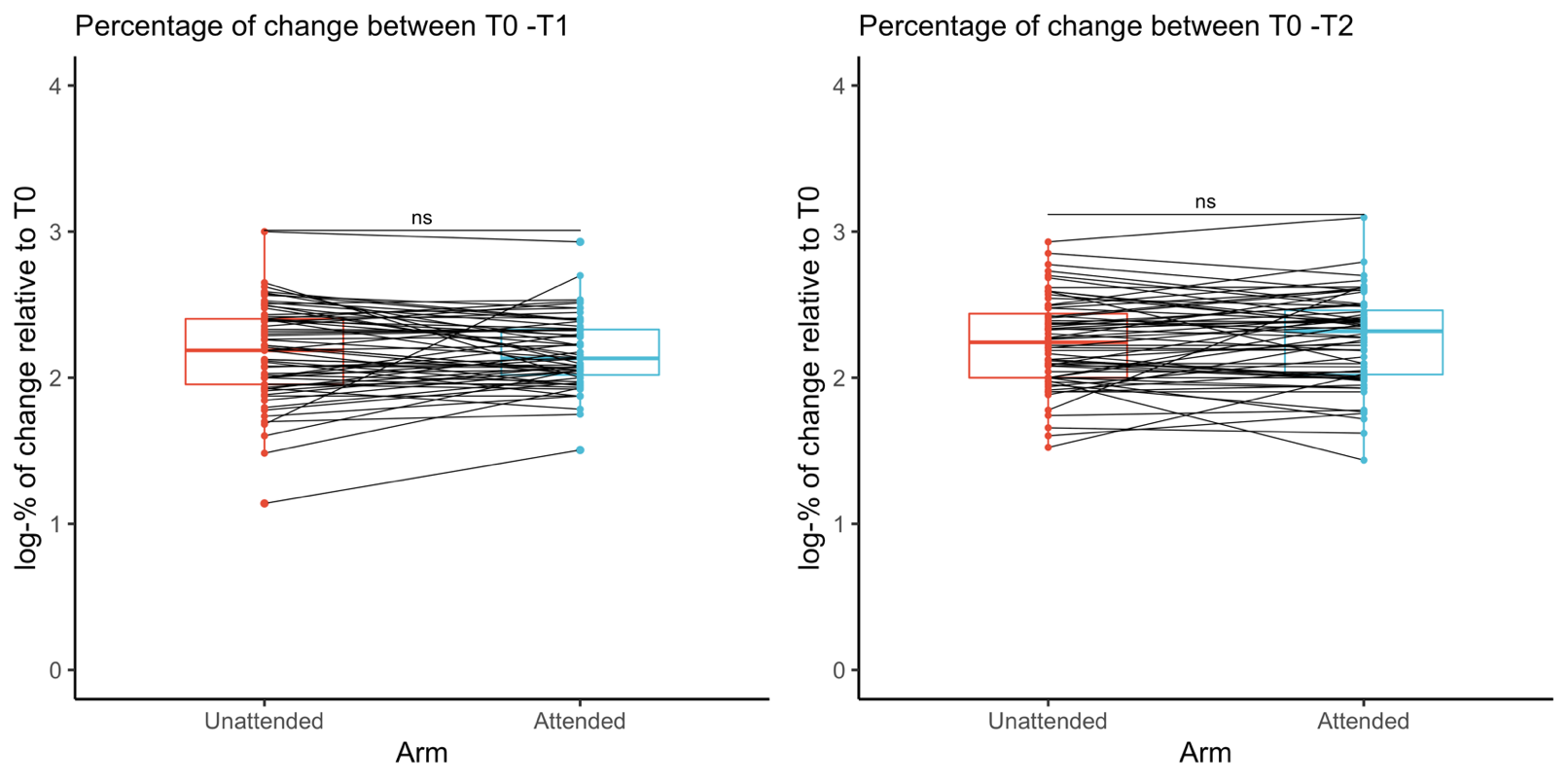


(A)

(B)

**Supplementary Figure 4 | Unattended and attended arm compared in terms of percentage of change in mechanical pinprick sensitivity assessed using a probe with an exerting force of 64mN.**

**(A)** Percentage of change relative to T0 and T2, **(B)** percentage of change relative to T0 and T1. The red and the blue bold lines in the boxplots represents the median of the mechanical pinprick sensitivity in terms of percentage of change, the red one for the unattended arm and the blue one for the attended arm. Each dot represents a single participants perceived intensity in terms of percentage of change, for each time points T1 (A) and T2 (B), relative to T0. The continuous line indicated with “ns” indicates that there was not significant difference between the two conditions, attended and unattended.

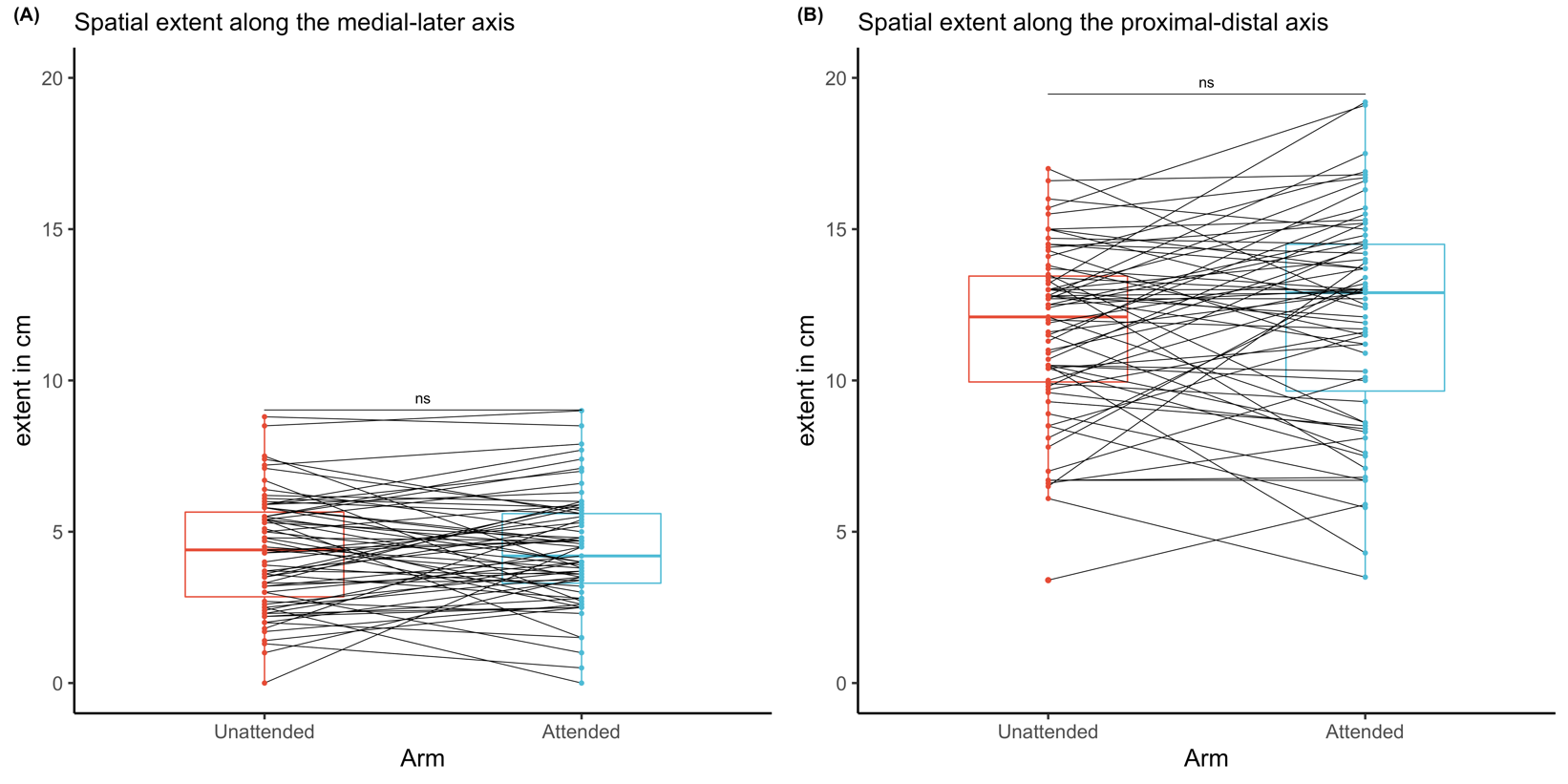


**Supplementary Figure 5 | ﻿Spatial extent of increased pinprick sensitivity induced by high frequency stimulation (HFS) at the attended**

**and the unattended arm, 20 min after HFS, assessed along the (A) Medial-later axis and (B) Proximal-distal axis,**

**assessed using a probe with an exerting force of 64mN.**

The red and the blue bold lines in the boxplots represents the median of the extent of the area of increased mechanical sensitivity, the red one for the unattended arm and the blue one for the attended arm. Each dot represents a single participants extent of the area of increased sensitivity along the two different axes, (A) for medial-lateral, and (B) for proximal-distal.

The continuous line indicated with “ns” indicates that there was not significant difference between the two conditions, attended and unattended

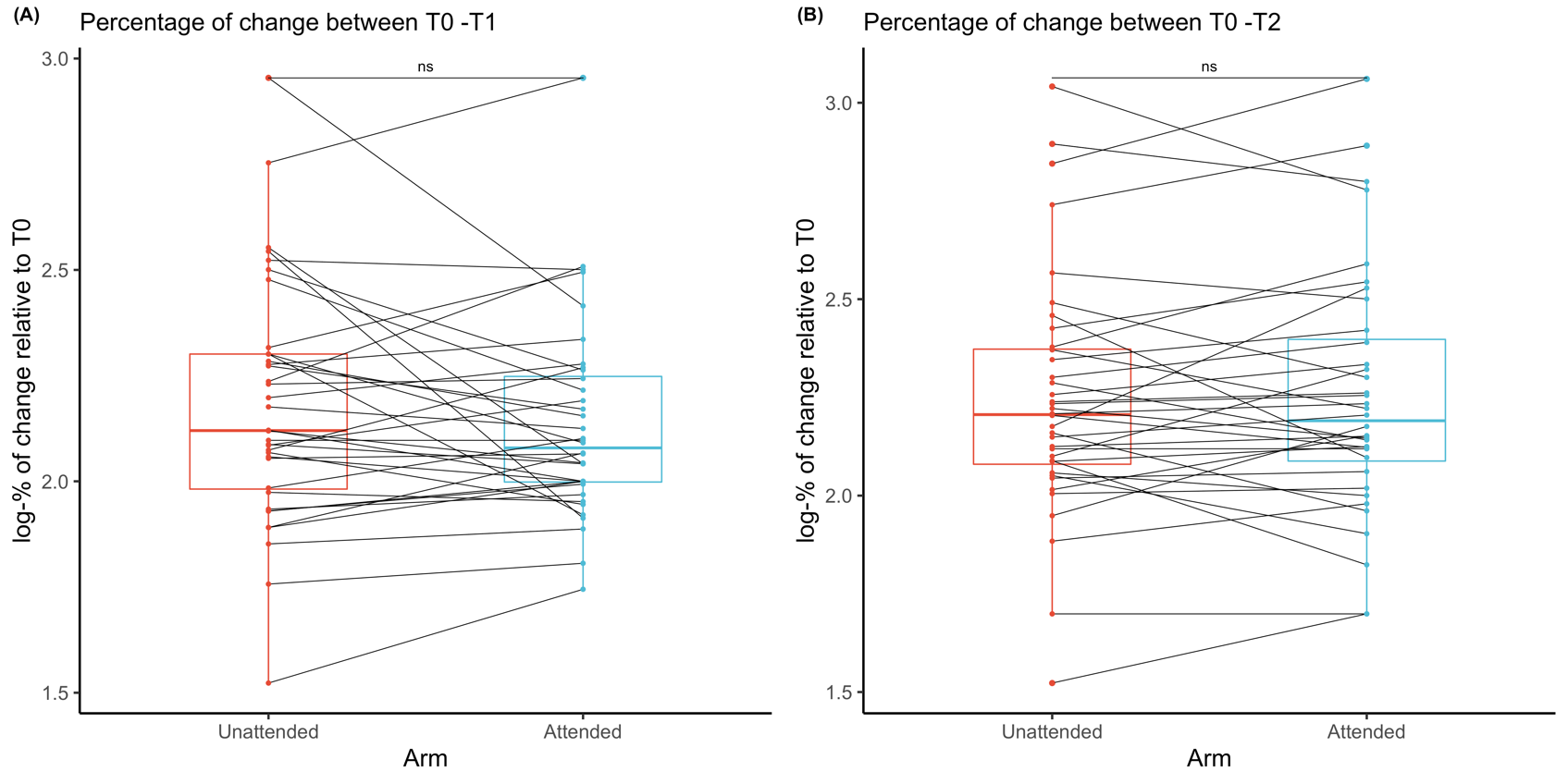


**Supplementary Figure 6 | ﻿The unattended and the attended arm from 36 participants able to detect at least 5 out of 10 target stimuli with less than 10 false alarms compared in terms of percentage of change in mechanical pinprick sensitivity assessed using a probe with an exerting force of 128mN.**

**(A) Medial-later axis and (B) Proximal-distal axis.**

The red and the blue bold lines in the boxplots represents the median of the mechanical pinprick sensitivity in terms of percentage of change, the red one for the unattended arm and the blue one for the attended arm. Each dot represents a single participants perceived intensity in terms of percentage of change, for each time points T1 (A) and T2 (B), relative to T0. The continuous line indicated with “ns” indicates that there was not significant difference between the two conditions, attended and unattended.

The percentages of change of mechanical sensitivity assessed with the 128 mN pinprick stimuli, were not statistically different between the attended and unattended arms, neither at T1 **(A)** (attended arm: 2.13 ± .234; unattended arm: 2.18 ± .28; t(35)= -1.19, d= -.199, p = .87), nor at T2 **(B)** (attended arm: 2.25 ± .31; unattended arm: 2.24 ± .30; t(35)= .282, d =.0469, p = .39).

**
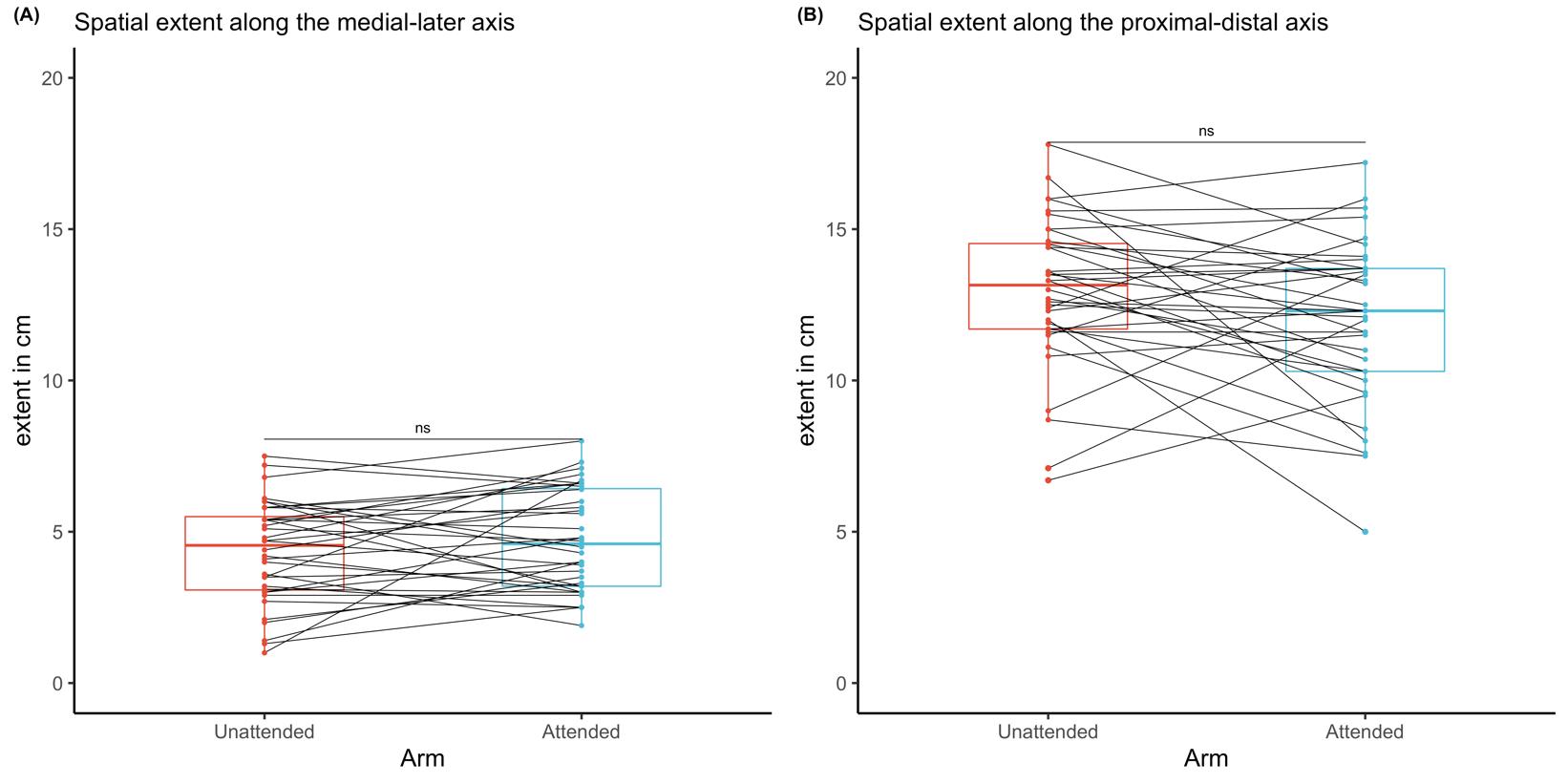
**

**Supplementary Figure 7 | ﻿The unattended and the attended arm from 36 participants able to detect at least 5 out of 10 target stimuli with less than 10 false alarms compared in terms of spatial extent in increased mechanical pinprick sensitivity assessed using a probe with an exerting force of 128mN along**

**(A) Medial-later axis, and (B) Proximal-distal axis.**

The red and the blue bold lines in the boxplots represents the median of the extent of the area of increased mechanical sensitivity, the red one for the unattended arm and the blue one for the attended arm. Each dot represents a single participants extent of the area of increased sensitivity along the two different axes, (A) for medial-lateral, and (B) for proximal-distal.

The continuous line indicated with “ns” indicates that there was not significant difference between the two conditions, attended and unattended. The length of the medial-later axis was not significantly different on the attended arm (4.72 ± .1.68) than the one on the unattended arm (4.34 ± 1.68; t (35) = 1.37, d= .229, p= .089). The same was observed for the proximal-distal axis. There was no statistical difference between the length of the proximal-distal axis on the attended arm (12 ± 2.67) as compared to the length on the unattended arm (12.9 ± 2.47; t (35) = -1.91, d =-.319, p= .96).


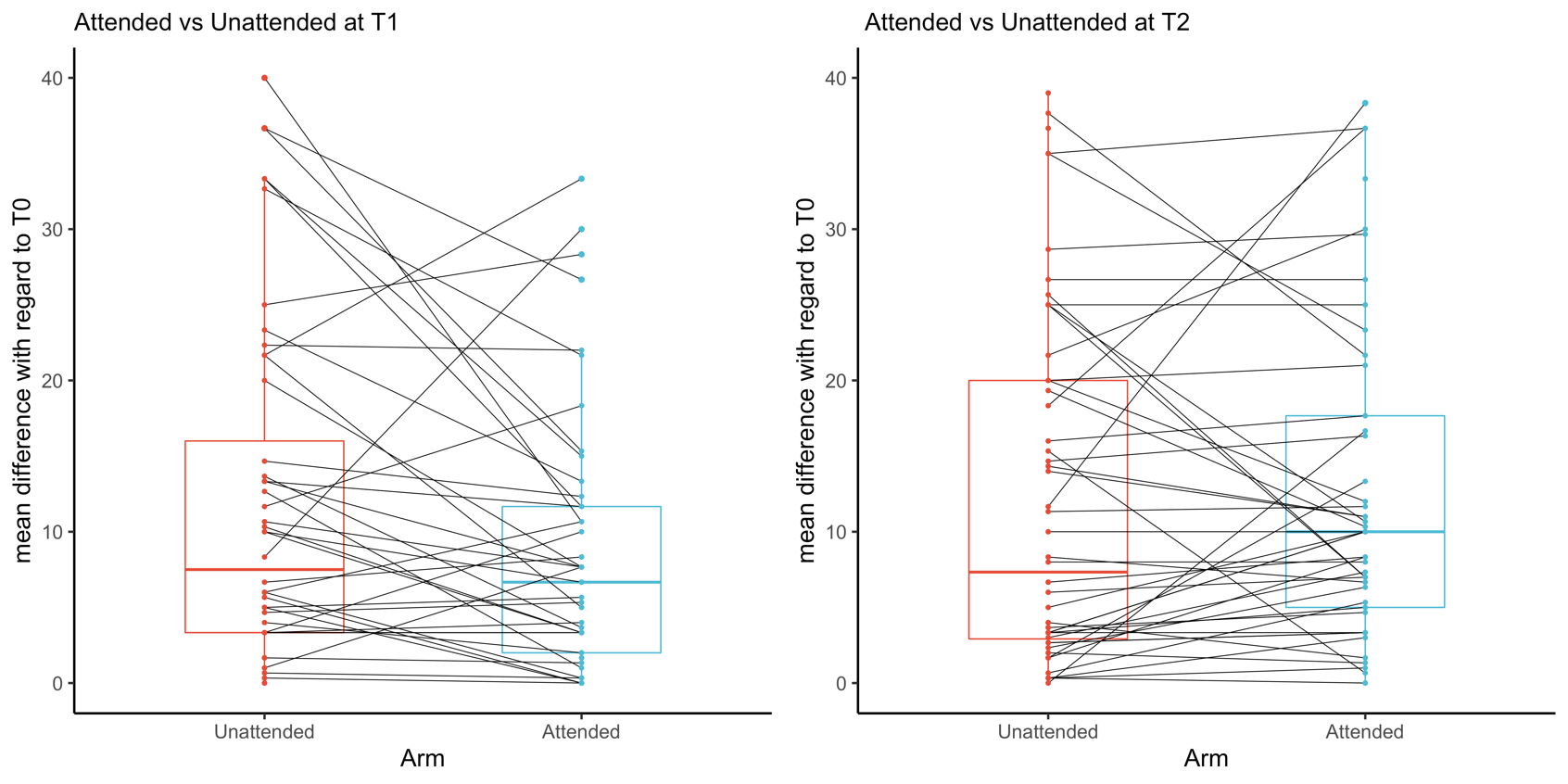


(A)

(B)

ns

ns

**Supplementary Figure 8 | Increased mechanical sensitivity induced by the high-frequency stimulation (HFS) between ﻿the unattended and the attended arm, in terms of mean difference between regarding T0, (A) immediately after the induction of high-frequency stimulation, and (B) 20 minutes later, assessed using a probe with an exerting force of 128mN.**

The red and the blue bold lines in the boxplots represents the median of the mechanical pinprick sensitivity in terms of percentage of change, the red one for the unattended arm and the blue one for the attended arm. Each dot represents a single participants mean difference, for each time points T1 (A) and T2 (B), relative to T0. The continuous line indicated with “ns” indicates that there was not significant difference between the two conditions, attended and unattended.

The mean difference in mechanical sensitivity assessed with the 128 mN pinprick stimuli, were not statistically different between the attended and unattended arms, neither at T1 **(A)** (attended arm: 5.4± 9.8; unattended arm: 7.9 ± 13.4; t (65) = -2.13, d= -.262, p= .98), nor at T2 **(B)** (attended arm: 10.9±15; unattended arm: 11.6 ±15.0; t (65) = -0.60, d= -0.07, p= .72).
